# Antibody–nanobody combination increases their neutralizing activity against SARS-CoV-2 and nanobody H11-H4 is effective against Alpha, Kappa and Delta variants

**DOI:** 10.1038/s41598-022-14263-1

**Published:** 2022-06-11

**Authors:** Hung Nguyen, Mai Suan Li

**Affiliations:** 1 Institute of Physics, Polish Academy of Sciences, Al. Lotnikow 32/46, 02-668 Warsaw, Poland; 2 Life Science Lab, Institute for Computational Science and Technology, Quang Trung Software City, Tan Chanh Hiep Ward, District 12, Ho Chi Minh City, Vietnam

**Keywords:** Biophysics, Computational biology and bioinformatics

## Abstract

The global spread of COVID-19 is devastating health systems and economies worldwide. While the use of vaccines has yielded encouraging results, the emergence of new variants of SARS-CoV-2 shows that combating COVID-19 remains a big challenge. One of the most promising treatments is the use of not only antibodies, but also nanobodies. Recent experimental studies revealed that the combination of antibody and nanobody can significantly improve their neutralizing ability through binding to the SARS-CoV-2 spike protein, but the molecular mechanisms underlying this observation remain largely unknown. In this work, we investigated the binding affinity of the CR3022 antibody and H11-H4 nanobody to the SARS-CoV-2 receptor binding domain (RBD) using molecular modeling. Both all-atom steered molecular dynamics simulations and coarse-grained umbrella sampling showed that, consistent with the experiment, CR3022 associates with RBD more strongly than H11-H4. We predict that the combination of CR3022 and H11-H4 considerably increases their binding affinity to the spike protein. The electrostatic interaction was found to control the association strength of CR3022, but the van der Waals interaction dominates in the case of H11-H4. However, our study for a larger set of nanobodies and antibodies showed that the relative role of these interactions depends on the specific complex. Importantly, we showed Beta, Gamma, Lambda, and Mu variants reduce the H11-H4 activity while Alpha, Kappa and Delta variants increase its neutralizing ability, which is in line with experiment reporting that the nanobody elicited from the llama is very promising for fighting against the Delta variant.

## Introduction

**Figure 1:**
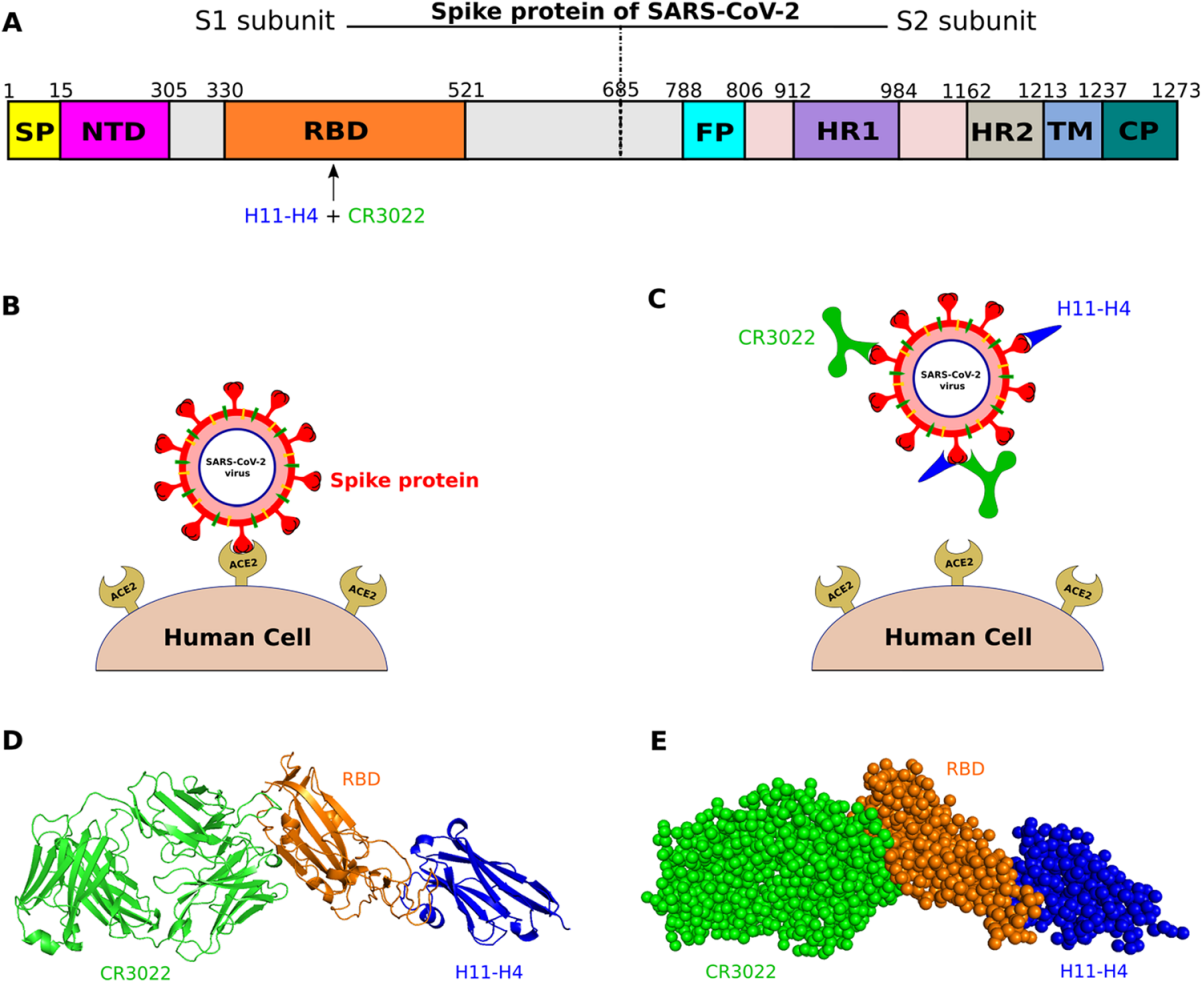
(**A**) Schematic description of the S protein of SARS-CoV-2, which consists of the S1 and S2 subunits. (**B**) SARS-CoV-2 S protein binds to human ACE2 before entering cells. (**C**) H11-H4 and CR3022 bind to S protein, preventing the virus from entering cells. The 3D structures of H11-H4 and CR3022 bound to RBD are shown in all-atom (**D**) and coarse-grained (**E**) models.

Fully human monoclonal antibodies (mAbs) have recently been demonstrated to be a promising class of therapeutics against severe acute respiratory syndrome coronavirus 2 (SARS-CoV-2) infection^1^. Several studies have shown that convalescent plasma from recovered SARS-CoV-2 patients, which contains neutralizing antibodies generated by an adaptive immune response, can effectively improve patient survival rate^2–4^. However, plasma-based therapies cannot be produced on a large scale. Thus, the search for potent antibody therapies on an industrial-scale is becoming one of the most feasible strategies for combating SARS-CoV-2. Spike (S) protein of SARS-CoV-2 (Fig. 1A), a multi-functional molecular machine that binds to angiotensin-converting enzyme 2 (ACE2) of the human cell (Fig. 1B), is a target of neutralizing antibodies and is the focus of therapeutic and vaccine development efforts^5^.

S protein consists of N-terminal S1 and C-terminal S2 subunits^6,7^ (Fig. 1A) that have a function to mediate receptor binding and membrane fusion^6,8^. Especially, both the receptor-binding domain (RBD) and the N-terminal domain (NTD) in the S1 subunit are important for determining host ranges and tissue nutrition^9,10^. NTD is able to recognize specific sugar components during the initial association of the virus and host cells^11,12^ and is critical in the transition of the S protein from pre-fusion to post-fusion^13,14^.

RBD binding to human cells is a critical step, allowing coronaviruses to enter cells and cause infection^15,16^. The S2 subunit contains heptad repeat region 1 (HR1) and 2 (HR2), both of which interact to form a six-helix bundle (6-HB) fusion core structure that brings the viral and cell target membranes into close proximity for fusion. Peptide fusion (FP) targeting the HR1 and HR2 regions is considered as a key factor for developing broad-spectrum viral fusion to inhibit t6-HB formation and virus-cell membrane fusion^17^. Therefore, RBD and NTD from the S1 subunit and FP from the S2 subunit of protein S may serve as important therapeutic targets against SARS-CoV-2 infection.

Antibodies that can neutralize SARS-CoV-2 can bind to RBD, NTD or FP, but most of them have been found to bind with RBD^18,19^, making RBD a key target. Due to different experimental methods, conditions and calibrations, recent studies have provided biased results regarding the binding affinity of antibodies, which has hampered the development of antibody-based therapy for SARS-CoV-2^18^. For instance, according to Tian et al.^20^, antibody CR3022, derived from a convalescent SARS-CoV-2 patient may be active due to its strong binding to RBD with a dissociation constant K_d_ = 6.3 nM, but another study reported that this is not the case, since the corresponding K_d_ is much higher (K_d_ = 115 nM) (Table 1)^21^.

**Table 1:** Dissociation constant K_d_ (nM) obtained by in vitro experiment.

	K_d_, *∆G*_exp_ (experiment)	*∆G*_bind_ (our simulation)
H11-H4–RBD	K_d_ = 11.8 ± 1.5 nM (Huo et al*.*^25^) ∆G_exp_ = − 10.9 ± 0.1 kcal/mol	− 19.8
CR3022–RBD	K_d_ = 6.3 nM (Tian et al*.*^20^) ∆G_exp_ = − 11.3 kcal/mol OR K_d_ = 115 ± 3.0 nM (Yuan et al*.*^21^) ∆G_exp_ = − 9.5 ± 0.02 kcal/mol	− 21.4
H11-H4 + CR3022–RBD	N/A	− 23.9

The experimental binding free energy *∆G*_exp_ was converted from K_d_ using ${\Delta G}_{exp}=RTln{K}_{d}$. Binding free energy ∆G_bind_ (kcal/mol) was obtained using coarse-grained umbrella sampling and Eq. (4) for the H11-H4–RBD, CR3022–RBD, and H11-H4 + CR3022–RBD complexes. Shown is the WT case .

Nanobodies are small, but stable and straightforward to manufacture. They serve as an alternative to conventional antibodies as diagnostic and structural biology tools^22^, and have recently been developed as therapeutic agents against SARS-CoV-2^23,24^. H11-H4, a llama-derived nanobody binds to RBD with K_d_ = 11.8 nM^25^ (Table 1), which is greater than K_d_ obtained by Tian et al*.*^20^ for CR3022, suggesting that H11-H4 binds to RBD weaker than the CR3022 antibody. However, when comparing with K_d_ reported by Yuan et al.^21^ (Table 1), we see that H11-H4 binds to RBD more strongly than CR3022. To solve this dispute we will calculate binding affinity using molecular simulation.

It is important to note that nanobodies can be used alone or in combination with antibodies in the treatment of severely ill patients with Covid-19 (Fig. 1C)^25^. The binding affinity of antibodies to SARS-CoV-2 was computationally studied^26,27^, but the binding free energy of nanobodies has not been calculated although their interaction with RBD was explored using molecular modeling. Moreover, the effect of the combination of antibodies and nanobodies on their neutralizing ability has not been theoretically investigated. Therefore, in this paper, using the coarse grained model and umbrella sampling, we will calculate the binding free energy of the H11-H4 nanobody with RBD and study how the combination of the CR3022 antibody and the H11-H4 nanobody changes their ability to neutralize SARS-CoV-2.

There have been many experimental studies of SARS-CoV-2 variants such as Alpha, Beta, Gamma, Kappa, Delta, Lambda, Mu, etc.^28–36^, which reduce the neutralizing ability of most antibodies and nanobodies against SARS-CoV-2^37–39^. The Beta variant reduces the neutralizing potential of antibodies REGN10933, C105, BD23 and H11-H4 nanobodies, etc^38^. However, recent studies have identified some potential antibodies and nanobodies that can effectively neutralize most of these variants^40,41^. For instance, cocktails of antibodies REGN10933 and REGN10987 can neutralize the Lambda variant^40^, while nanobodies obtained from the llama are good agents against the Delta variant^41^. In this study, we use steered molecular dynamics (SMD) to access the binding affinity between H11-H4 and the SARS-CoV-2 variants, including Alpha, Beta, Gamma, Kappa, Delta, Lambda and Mu variants. We show that H11-H4 can effectively neutralize Alpha and Delta variants, which makes it a very promising therapy for Covid-19.

## Material and methods

### PDB structures of the three studied systems

The structures of H11-H4–RBD, CR3022–RBD, and H11-H4 + CR3022–RBD complexes were extracted from the Protein Data Bank with PDB ID: 6ZH9^25^. Modeler package^42^ was used to add the missing residues. The structure of H11-H4 + CR3022–RBD complex is shown in Fig. 1D (all-atom) and Fig. 1E (coarse-grained) prepared by using the PyMOL package^43^. All mutations including variants Alpha, Beta, Gamma, Kappa, Delta, Lambda and Mu were generated by using the mutagenesis tool in PyMOL package.

### All-atom molecular dynamics simulations

All-atom molecular dynamics (MD) simulations were performed using the CHARMM36M force field^44^ implemented in the GROMACS 2016 package^45^ at 310 K and isotropic pressure of 1 bar, which was obtained using the v-rescale^46^ and Parrinello-Rahman^47^ algorithms, respectively. The water model TIP3P^48^ was used for all systems. Bond lengths were constrained by the linear constraint solver (LINCS) algorithm^49^, allowing a time step of 2 fs.

Electrostatic and van der Waals interactions were calculated with a cutoff of 1.4 nm, and the non-bonded interaction pair-list was updated every 10 fs. The Particle Mesh Ewald algorithm^50^ was used to treat long-range electrostatic interactions. Periodic boundary conditions were applied in all directions. The energy of the system was first minimized by using the steepest-descent algorithm, then a short 3 ns MD simulation was performed in the NVT and NPT ensembles. Production MD simulation of 100 ns was performed with the help of the leap-frog algorithm^51^. For each complex, using the “gmx_mpi cluster” tool available in GROMACS, we grouped the snapshots collected from the 100 ns of conventional MD simulation into clusters. We then selected 5 representative structures from the five most populated clusters and used them as the initial configuration for running 5 trajectories of steered molecular dynamics (SMD) simulations^52–55^.

#### Steered molecular dynamics

**Figure 2:**
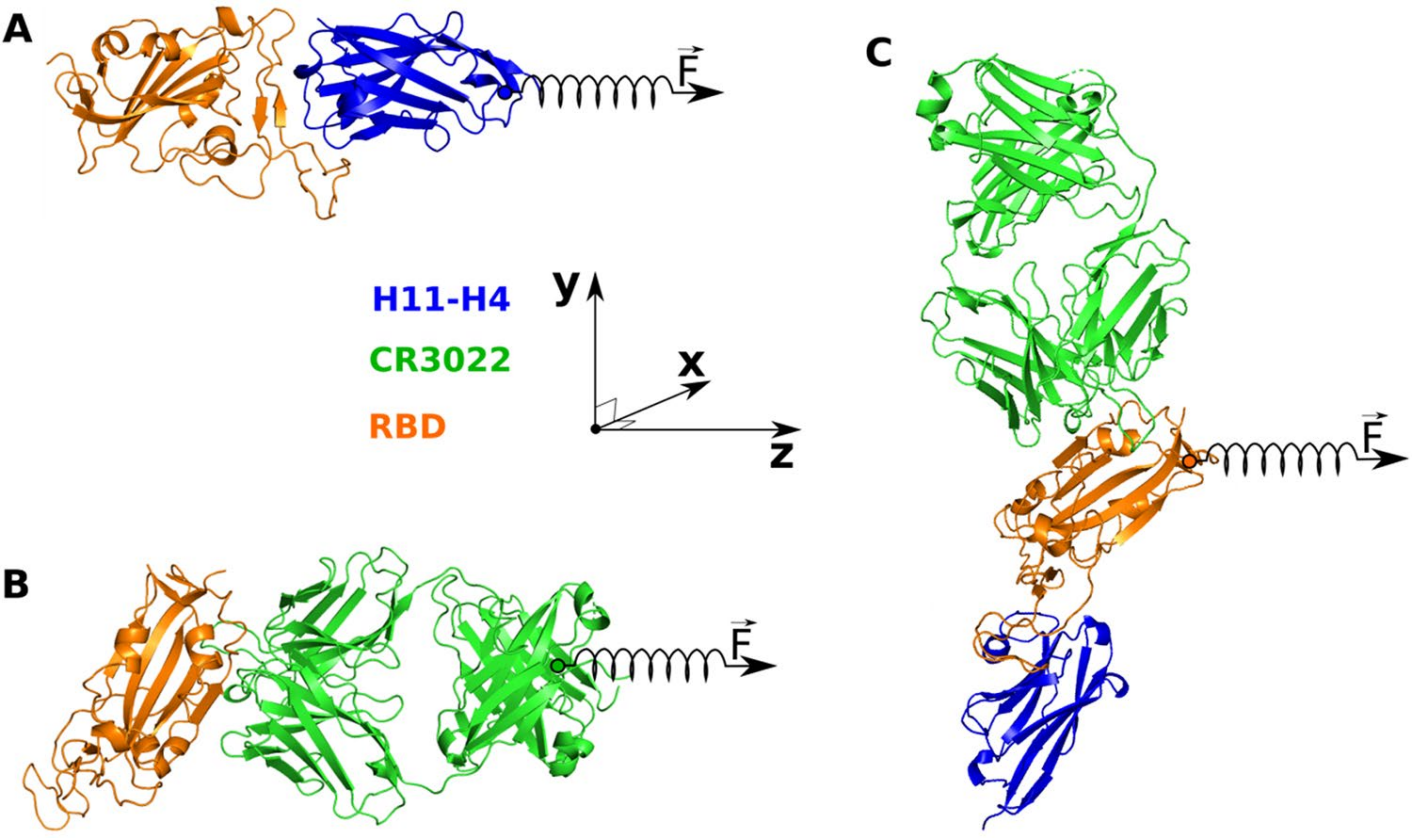
Structure of H11-H4 + CR3022–RBD retrieved from PDB with ID 6ZH9. RBD is shown in orange, while green and blue describe CR3022 and H11-H4. (**A**) H11-H4–RBD complex, external force $\overrightarrow{F}$ is applied to the H11-H4 nanobody through a dummy atom connected to a spring. (**B**) CR3022–RBD complex, $\overrightarrow{F}$ is applied to the CR3022 antibody. (**C**) H11-H4 + CR3022–RBD complex, $\overrightarrow{F}$ is applied to RBD. The pulling direction in SMD simulations is shown with a spring along the z-axis.

We carried out SMD simulations to pull H11-H4 or CR3022 from the binding region of RBD as well as pulling RBD from the binding region of H11-H4 and CR3022 (Fig. 2). In the case of H11-H4–RBD and CR3022–RBD, an external force is applied to a dummy atom, which is linked to the Cα atom closest to the center of mass (COM) of H11-H4 or CR3022. The pulling direction is parallel to the vector connecting COMs of RBD and nanobody or antibody (Fig. 2A,B). In order to prevent RBD from drifting under the action of an external force, its backbone was restrained, but the side chain could fluctuate. The choice of pulling direction is different in the case of H11-H4 + CR3022–RBD due to the presence of three molecules. In this case an external force is applied to a dummy atom that is bonded to the Cα atom closest to the COM of RBD, and the pulling direction is along the line connecting The RBD COM in perpendicular to the line connecting the COMs of H11-H4 and CR3022 (Fig. 2C). During the SMD simulation the backbone of H11-H4 and CR3022 was restrained. For convenience, three complexes H11-H4–RBD, CR3022–RBD and H11-H4 + CR3022–RBD were rotated so that the pulling direction was always along the z-axis (Fig. 2).

One of the limitations of unidirectional pulling is that not all rotational states of proteins can be sampled. However, as shown in previous works^26,55^, this approach provides reasonable results on the relative binding affinity of protein–protein complexes.

The pulling force experienced by a stretched molecule is calculated as follows:

1 $$F=k\left(\Delta z-vt\right)$$

where *k* is the stiffness of the spring, *v* is the pulling velocity, $\Delta z$ is the displacement of a real atom connected to the spring in the direction of pulling, respectively. The spring constant *k* was set to 600 kJ/(mol nm^2^) (≈ 1020 pN/nm), which is a typical value used in atomic force microscopy (AFM) experiments^56^.

Using the force–displacement profile obtained from SMD simulations, the non-equilibrium work (*W*) performed by the pulled chain (H11-H4, CR3022 or H11-H4 + CR3022) was estimated using the trapezoidal rule:

2 $$W=\int Fdz={\sum }_{i=1}^{N}\frac{{F}_{i+1}+{F}_{i}}{2}\left({z}_{i+1}-{z}_{i}\right)$$

where N is the number of simulation steps, *F*_*i*_ and z_*i*_ are the force determined by Eq. (1) and the position at step *i*, respectively.

To estimate the binding free energy (*∆G*), we can use Jarzynski’s equality^57^ extended to the case when the applied external force grows at a constant speed *v*^58^:

3 $$exp\left(\frac{-\Delta G}{{k}_{B}T}\right)={\left \langle exp\left(\frac{{W}_{t}-\frac{1}{2}k{\left({z}_{t}-vt\right)}^{2}}{{k}_{B}T}\right) \right \rangle }_{N}$$

here ${\langle \dots \rangle }_{N}$ is the average over *N* trajectories, ${z}_{t}$ is the time-dependent displacement, and *W*_*t*_ is the non-equilibrium work at time *t* determined by Eq. (2).

From Eq. (3), we can extract the equilibrium free energy if the number of SMD trajectories is large enough and the pulling is sufficiently slow. Therefore, this approach is practical for small systems^59^ but not for large systems such as those studied in this work. However, we can estimate the non-equilibrium binding and unbinding barriers separating the transition state (TS) from the bound state at *t* = 0 and the unbound state at *t*_end_^60^, which allows us to discern weak binding from strong binding.

Rectangular boxes with dimension of 10 × 9 × 25 nm^3^, 10 × 11 × 25 nm^3^ and 10 × 18 × 25 nm^3^ were used for H11-H4–RBD, CR3022–RBD and H11-H4 + CR3022–RBD, respectively. The complexes were immersed in a 0.15 M sodium chloride salt solution and counter ions were added to neutralize the system. In order to show that our results on the relative binding affinity is independent of the pulling speed, for each system, 5 different trajectories were run at *v* = 0.5 and 1 nm/ns.

### Coarse-grained simulation

Since the combination of SMD with Jarzynski’s equality does not allow us to calculate the equilibrium binding free energy, we will use umbrella sampling (US). However, this approach is very time consuming if we use all-atom models, because in our case the proteins and antibodies are large. Therefore, we used the MARTINI 2.2 force field developed for coarse-grained (CG) modeling of biological systems such as biological membrane, protein, nucleotide, and etc^61–63^. This force field is accurate enough for extracting the interaction energy for a pair of proteins in an aqueous environment from constraint force profiles. The standard MARTINI water model was used with a minimum distance between water beads of 1.0 nm^64^. The system was neutralized by adding sodium chloride salt solution. The temperature was set at T = 300 K with a Berendsen thermostat, and pressure was set at p = 1.0 bar with a Berendsen barostat^65,66^. Bond lengths in the aromatic amino acid side chains and the bonds between the backbone and side chains were constrained by the LINCS algorithm^49^.

To perform coarse-grained umbrella sampling^67^ (CG-US) simulations, we made a series of configurations along the z-axis involving 81 windows each of 0.1 nm (Fig. S1). Here *z* is the reaction coordinate (RC). The choice of the z-axis has been already described in the SMD method. Namely, for CR3022-RBD and H11-H4-RBD this axis connects two COMs (Fig. S1A,B), while for H11-H4 + CR3022-RBD it is parallel to the line connecting COMs of H11-H and CR3022 (Fig. S1C).

To create an initial configuration for the first window, energy minimization was performed and the neutralized and solvated structure was simulated for 1 ns with position restraints throughout the structure to allow the solvent to equilibrate around the solute. Temperature and pressure were relaxed for 10 ns. The resulting conformation was then used as the initial conformation in a subsequent 100 ns run without position restraints. The last snapshot obtained in this run will be used as the initial configuration for the first window in CG-US simulations.

To generate the initial configuration for other windows, we pulled antibody, nanobody or RBD to the corresponding window. Then we performed energy minimization and equilibration using a 5 ns MD simulation restraining the distance between COMs of subsystems. The last snapshot obtained in this simulation will be used as an initial conformation for the production run.

To hold one chain (H11-H4, CR3022 or RBD) around the center of each window, we applied a bias harmonic potential with a spring constant of 600 kJ/mol/nm^2^ to make sure that the interacting surface of both targets is not change. To get a good sampling, for each window, we performed a conventional MD production run of 1000 ns. The WHAM procedure^68^ is then used to determine a one-dimensional potential of mean force (1D PMF) as a function of the reaction coordinate *z*.

The binding free energy ($\Delta {G}_{bind}$) is defined as the difference between the free energies in the bound and unbound states^69^:

4 $$\Delta {G}_{bind}=\left(-{k}_{B}Tln{\int }^{bound}{e}^{\frac{-{G}_{1D}\left(z\right)}{{k}_{B}T}}\right)-\left({-k}_{B}Tln{\int }^{unbound}{e}^{\frac{-{G}_{1D}\left(z\right)}{{k}_{B}T}}\right)$$

here ${G}_{1D}\left(z\right)$ is the 1D PMF as a function of *z*, *k*_*B*_ is the Boltzmann constant, and *T* is the absolute temperature. Symbols ∫^*bound*^ and ∫^*unbound*^ refer to summation over bound and unbound regions, respectively. To determine the cut-off distance between the bound and unbound states we calculated the number interchain contacts as a function of the distance between pulled and nonpulled chains in CG-US simulations. Then the cutoff distance is the distance above which interchain contacts disappear (Fig. S2).

### Measures used in data analysis

A hydrogen bond (HB) is formed if the distance between donor D and acceptor A is less than 0.35 nm, the H-A distance is less than 0.27 nm, and the D-H-A angle is greater than 135 degrees. A non-bonded contact (NBC) between two residues is formed if the shortest distance between their atoms is within 0.39 nm. 2D contact networks of HBs and NBCs of CR3022–RBD and H11-H4–RBD were displayed using the LIGPLOT package^70^. The standard deviation (Er) are approximately expressed as follows:

5 $${E}_{r}=\sqrt{\frac{\sum_{i=1}^{N}{({x}_{i}-\langle x\rangle )}^{2}}{N-1}}$$

where N is the total number of data points in the data set, ${x}_{i}$ is the individual value of the *ith* in the data set, and $\langle x\rangle$ is the mean value of the data set.

## Results and discussion

### Hydrogen bond and non-bonded contact networks of CR3022-RBD and H11-H4-RBD complexes: analysis based on the PDB structure

Using the 6ZH9 PDB structure, we build networks of hydrogen bonds (HBs) and non-bonded contacts (NBCs) of H11-H4 and CR3022 with RBD (Fig. S3A–D). The numbers of H11-H4 and CR3022 residues that form HB and NBC with RBD are 11 and 19, respectively. There are 9 and 10 HBs for H11-H4-RBD and CR3022-RBD, respectively, while the numbers of NBCs of H11-H4-RBD and CR3022-RBD correspond to 14 and 20. The number of HBs and NBCs in the crystal structure cannot determine the binding affinity, since other factors also matter. However, more HBs and NBCs may indicate higher binding affinity, which suggests that CR3022 has a higher binding affinity for RBD than H11-H4. To verify this we will carry out SMD and coarse-grained umbrella simulations.

### Binding affinity of H11-H4 and CR3022 to RBD: SMD results

#### CR3022 binds to RBD more strongly than H11-H4 and combination of antibody and nanobody enhances their neutralizing activity

**Figure 3:**
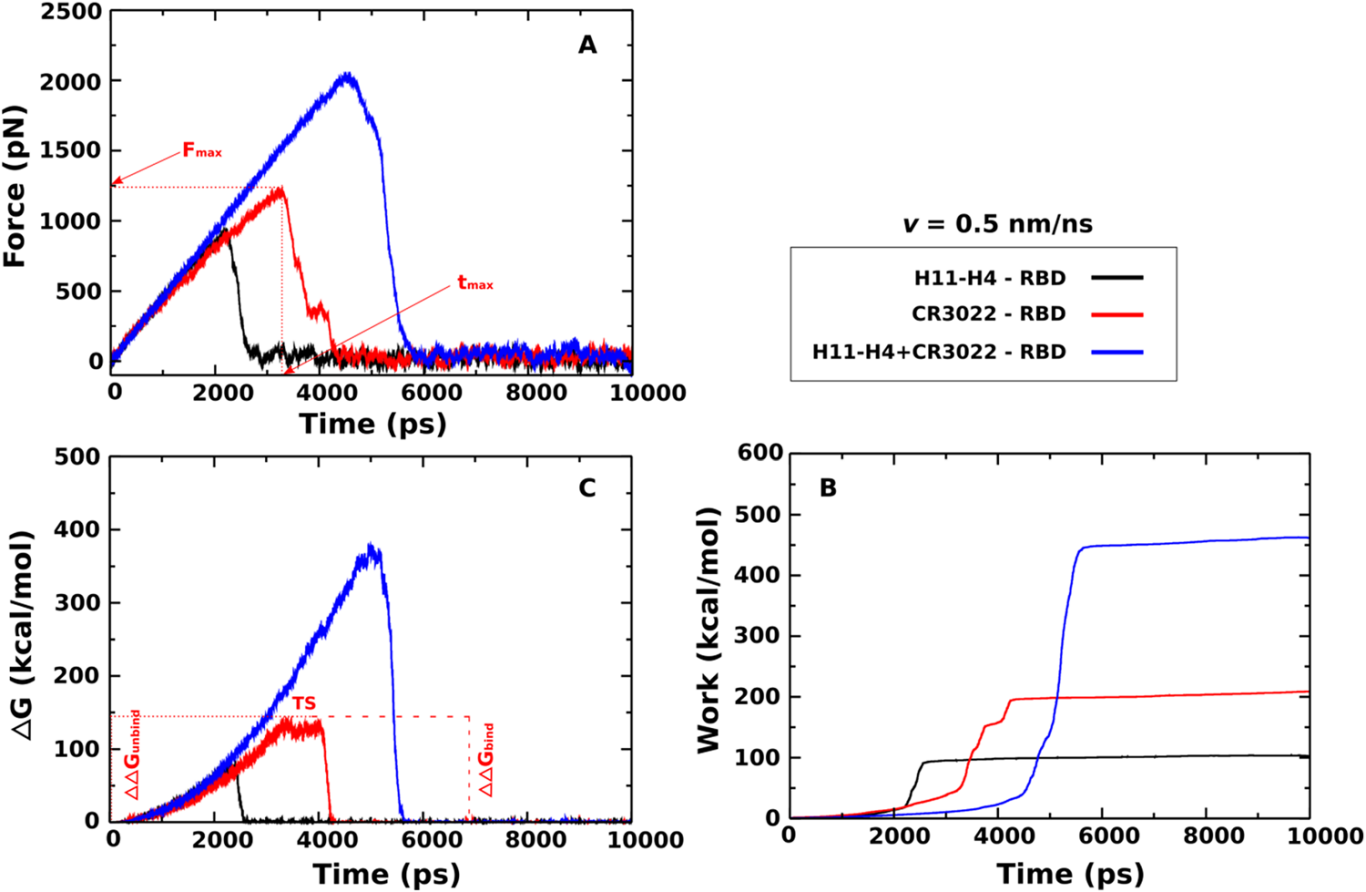
Time dependence of the pulling force (**A**), work (**B**), and (non-equilibrium energy profiles (**C**) of the H11-H4-RBD, CR3022-RBD, and H11-H4 + CR3022-RBD. The results were obtained at *v* = 0.5 nm/ns and averaged from five independent SMD runs.

Force–time profiles obtained at *v* = 0.5 nm/ns for the three complexes (Fig. 3A, Table 2) show that CR3022 (*F*_max_ = 1214.2 ± 21.2 kcal/mol) binds to RBD more strongly than H11-H4 (*F*_max_ = 925.6 ± 15.2 kcal/mol) to RBD. It should be noted that the rupture force *F*_max_ appears to be quite high due to the fast pulling. In the so-called Bell approximation, where the transition state separating the bound state from the unbound state is independent of external force, *F*_max_ ~ ln(*v*)^71^, where *v* is the puling speed. Beyond the Bell approximation, the dependence of *F*_max_ on *v* is more complex^72^.

**Table 2:** Rupture force (*F*_max_), rupture time (*t*_max_), work of the external force (*W*), and non-equilibrium binding free energy *∆G*_bind_ and *∆G*_unbind_ for the H11-H4–RBD, CR3022–RBD, and H11-H4 + CR3022–RBD complexes.

	*v* = 0.5 nm/ns		
	H11-H4	CR3022	H11-H4 + CR3022
F_max_ (pN)	925.6 ± 15.2	1214.2 ± 21.2	2034.9 ± 27.7
t_max_ (ps)	2219.3 ± 38.5	3203.4 ± 36.9	4539.8 ± 47.2
W (kcal/mol)	101.6 ± 3.4	208.6 ± 5.3	461.3 ± 5.7
*∆*∆G_unbind_ (kcal/mol)	82.5 ± 2.1	140.2 ± 2.9	379.0 ± 4.2
*∆*∆G_bind_ (kcal/mol)	82.1 ± 2.5	137.9 ± 3.7	378.5 ± 4.4

The results were obtained from five independent SMD trajectories for the WT case at a pulling speed of *v* = 0.5 nm/ns. The errors represent standard deviations.

As expected, *F*_max_ increases with increasing of pulling speed (Tables 2 and S1, Figs. 3A and S4A). The unbinding time *t*_max_ of CR3022–RBD is also longer than H11-H4–RBD, and this time decreases with increasing *v*. It is important to note that if RBD is simultaneously extracted from the CR3022 antibody and H11-H4 nanobody, then at *v* = 0.5 nm/ns, *F*_max_ = 2034.9 ± 27.7 kcal/mol is required, which is approximately twice as much as in CR3022–RBD and H11-H4–RBD. Therefore, the combination of nanobody and antibody is expected to increase the binding affinity for RBD, which increases their neutralizing activity.

Since the non-equilibrium work (*W*) is better than *F*_max_ for characterizing the relative binding affinity^73^, we will look at it in detail. *W* increased rapidly until the pulled molecule (H11-H4, CR3022 or RBD) left the binding region, reaching a stable value when two subsystems ceased to interact (Fig. 3B), and also increases with *v*^74^. For *v* = 0.5 nm/ns, we obtained *W* = 101.6 ± 3.4, 208.6 ± 5.3 and 461.3 ± 5.7 kcal/mol for H11-H4–RBD, CR3022–RBD, and H11-H4 + CR3022–RBD, respectively (Table 2). Therefore, similar to *F*_max_, our results obtained for *W* further support the fact that CR3022 is more active than H11-H4 and their combination increases their binding strength to RBD.

Figure 3C displays the time dependence of the non-equilibrium binding free energy (*∆G*) estimated from Eq. (3) for the three complexes at *v* = 0.5 nm/ns. The maximum corresponds to the transition state (TS) with *∆G* = *∆G*_TS_. We have *∆G*_bound_ = *∆G*(*t*_0_ = 0) ≈ 0 kcal/mol at the beginning of the bound state, while the unbound state occurs at the end of simulation *∆G*_unbound_ = *∆G*(*t*_end_) ≈ 0 kcal/mol. Thus, the binding and unbinding free energy energies (barriers), defined as *∆∆G*_bind_ = *∆G*_TS_ − *∆G*_unbound_ and *∆∆G*_unbind_ = *∆G*_TS_ − *∆G*_bound_, are roughly equal. *∆∆G*_unbind_ = 82.5 ± 2.1, 140.2 ± 2.9, and 379.0 ± 4.2 kcal/mol for H11-H4–RBD, CR3022–RBD, and H11-H4 + CR3022–RBD, respectively, and *∆∆G*_bind_ = 82.1 ± 2.5, 137.9 ± 3.7 and 378.5 ± 4.4 kcal/mol (Table 2). This provides further evidence that CR3022 binds to RBD more tightly than H11-H4, and the binding affinity is higher if both H11-H4 and CR3022 are combined.

To ensure that our result does not depend on the pulling speed, we also conducted SMD simulations for *v* = 1 nm/ns. Although *F*_max_, *W*, and the non-equilibrium binding free energy increase with increasing *v*, the main conclusion about the relative binding affinities of the three complexes remains the same (Fig. S4, Table S1).

Therefore, our SMD data indicate that CR3022 binds more strongly to RBD than H11-H4, which is consistent with the experiment of Tian et al*.*^20^ and Huo et al*.*^25^ (Table 1). Measuring K_d_, Yuan et al*.*^21^ reported that the binding affinity of CR3022 for RBD is lower than that reported by Huo et al*.*^25^ for H11-H4. From this point of view our result is in conflict with Yuan et al*.*^21^ and Huo et al*.*^25^ The discrepancy may be caused by different techniques used by the two groups. Namely, Yuan et al*.*^21^ used biolayer interferometry binding assays, while isothermal titration calorimetry was employed by Huo et al*.*^25^ The advantage of our computational study is that we used the same model to compare the relative binding affinity, giving us confidence that CR3022 is a better binder than H11-H4.

#### Binding of H11-H4 to RBD is driven by vdW interaction, but binding of CR3022 and H11-H4 + CR3022 is driven by electrostatic interactions

**Figure 4:**
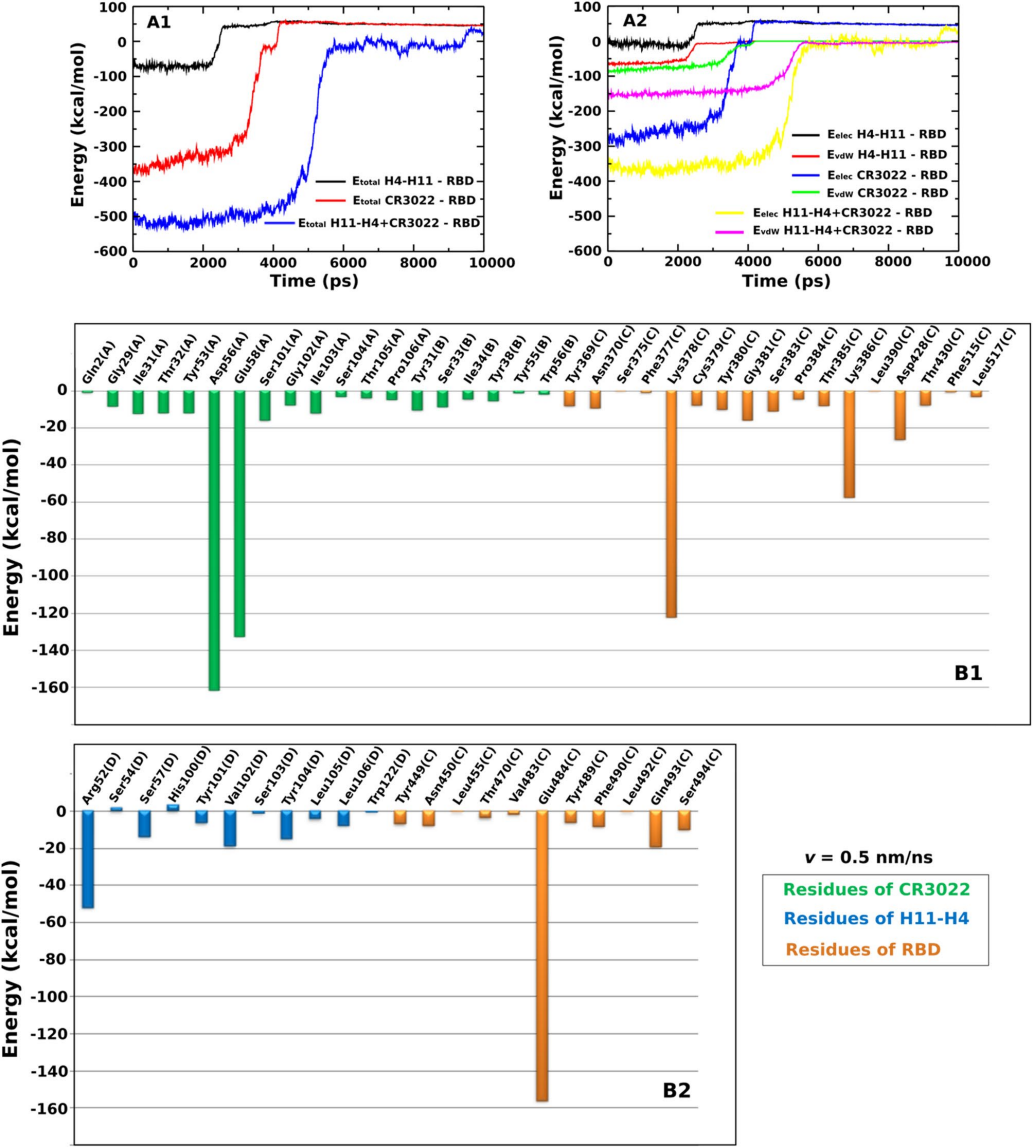
Time dependence of the total non-bonded interaction energy (sum of electrostatic and vdW) (**A1**) , and electrostatic and vdW interaction energies (**A2**) of the H11-H4-RBD, CR3022-RBD and H11-H4 + CR3022-RBD complexes. Total non-bonded interaction energy of residues located at the binding region of H11-H4-RBD (**B1**) and CR3022-RBD (**B2**). The results were obtained for a time window [0, *t*_max_] and averaged from five independent SMD runs at pulling speed *v* = 0.5 nm/ns.

A cutoff of 1.0 and 1.2 nm for van der Waals and electrostatic energies was applied to investigate the interaction of H11-H4–RBD, CR3022–RBD and H11-H4 + CR3022–RBD complexes. Fig. 4A1,A2 display the time dependence of the total non-bonded interaction energy *E*_total_, which is the sum of electrostatic (*E*_elec_) and van der Waals (*E*_vdW_) energies of H11-H4, CR3022, and H11-H4 + CR3022 interacting with RBD. These results were averaged over five SMD trajectories.

At *v* = 0.5 nm/ns, when bound, the *E*_elec_ of H11-H4–RBD, CR3022–RBD and H11-H4 + CR3022–RBD starts at a negative value, but in all three cases *E*_elec_ eventually reaches ≈ 50 kcal/mol in the unbound state. *E*_vdW_ of three complexes is also negative in the bound state reaching 0 kcal/mol in the unbound state (Fig. 4A2). Neglecting the contribution of entropy, the results shown in Fig. 4A1,A2 reaffirm the ordering of stability H11-H4 + CR3022 > CR3022 > H11-H4.

We calculated the mean interaction energy in the bound state by averaging over the time window [0, *t*_max_], where *t*_max_ is shown in Table 3. At *v* = 0.5 nm/ns, for CR3022–RDB, we obtained *E*_elec_ = − 252.8 ± 3.7 kcal/mol, which is clearly lower than *E*_vdW_ = − 77.1 ± 1.3 kcal/mol, implying that binding of CR3022 to RBD is driven by electrostatic interactions. This observation was also obtained previously^26^. The opposite occurs for the case of H11-H4, where the vdW interaction (*E*_*vdW*_ = − 61.8 ± 1.2 kcal/mol) is lower than the electrostatic interaction (*E*_*elec*_ = − 8.9 ± 0.7 kcal/mol), indicating that the vdW interaction dominates, but not the electrostatic interaction. Thus, the nature of binding of the H11-H4 nanobody is very different from CR3022 and the question of whether this remains true for other nanobodies is left for future research.

**Table 3:** Non-bonded interaction energies (kcal/mol) of the H11-H4–RBD, CR3022–RBD, and H11-H4 + CR3022–RBD complexes.

	*v* = 0.5 nm/ns		
	H11-H4–RBD	CR3022–RBD	H11-H4 + CR3022–RBD
E_vdW_	− 61.8 ± 1.2	− 77.1 ± 1.3	− 146.1 ± 1.1
E_elec_	− 8.9 ± 0.7	− 252.8 ± 3.7	− 355.5 ± 2.3
E_Total_	− 70.7 ± 1.9	− 329.9 ± 5.0	− 501.6 ± 3.4

The results were obtained for a [0-*t*_max_] time window and averaged from five SMD trajectories performed at a pulling speed of *v* = 0.5 nm/ns. The errors represent standard deviations.

If H11-H4 and CR3022 simultaneously bind to RBD, we obtained *E*_elec_ = − 355.5 ± 2.3 kcal/mol and *E*_vdW_ = − 146.1 ± 1.1 kcal/mol, which means that as in the single CR3022 case, the electrostatic interaction is more important than the vdW interaction in stabilizing the complex with RBD. The role of electrostatic and vdW interactions revealed in SMD simulations with *v* = 0.5 nm/ns remains unchanged for other pulling speed (*v* = 1 nm/ns) (Fig. S5, Table S2).

#### Role of specific residues in binding of H11-H4 and CR3022 to RBD

To understand the role of each residue at the interface (Fig. 4B1,B2) in stabilization of the three complexes, we calculated its interaction energy in the [0, *t*_max_] time window for pulling speed *v* = 0.5 nm/ns. For CR3022–RBD, residues Lys378(C), Lys386(C) and Asp428(C) of RBD, and residues Asp56(A) and Glu58(A) of CR3022 have the total non-bonded interactions smaller than − 20 kcal/mol (Fig. 4B1). For H11-H4–RBD, residues Glu484(C) and Gln493(C) of RBD and residue Arg52(D) of H11-H4 have the interaction energy smaller than − 20 kcal/mol (Fig. 4B2). Having a very low interaction energy of about − 156.3 kcal/mol, the Glu484(C) residue plays a very important role in the binding of the H11-H4 nanobody with the spike protein. Since the residue at position 484 is related to variants Beta (South Africa, lineage B.1.351, K417N, E484K, N501Y mutations), Gamma (Brazil, P.1 lineage, K417T, E484K, N501Y mutations), Kappa (India, B.1.617.1 lineages, L452R and E484Q mutations), and Mu (Colombia, B.1.621, R346K, E484K and N501Y), it is very interesting to consider these variants in more detail (see below).

#### The role of electrostatic and vdW interactions in the binding of nanobodies and antibodies to RBD depends on the specific system

Our previous work^26^ showed that the electrostatic interaction governs the binding of CR3022 to RBD, while in the present work the vdW interaction is found to be more important for H11-H4 nanobody. An interesting question emerges is if this conclusion is valid for other systems. To answer this question, we calculated the interaction energy for the ten antibody-RBD complexes and ten nanobody-RBD complexes using their PDB structures and the CHARMM36M force field with the TIP3P water model.

For antibodies, electrostatic interaction dominates over vdW interaction for the five antibodies, while vdW interaction takes over electrostatic interaction for the other five antibodies (Table S3). For nanobodies, the vdW interaction is more important than the Coulomb interaction in five cases, while the opposite occurs in the other four complexes. In the case of WNb 10-RBD, their role is almost the same (Table S4). Consequently, which interaction is dominant in the association of the antibodies and nanobodies with the spike protein depends on the specific system.

### Effects of mutations on binding affinity of H11-H4 to RBD: SMD results

As mentioned in the previous section, for the WT case, residue 484 makes an important contribution to the stability of the H11-H4–RBD complex. It has recently been demonstrated that this residue decreases the neutralizing activity of antibodies and nanobodies against the Covid-19 variants^37,41^ (see Table S5 for mutation points in some variants). To shed light on the molecular mechanisms underlying this interesting phenomenon we performed a series of SMD simulations at a pulling speed *v* = 0.5 nm/ns for the Alpha (United Kingdom, lineage B.1.1.7, N501Y), Beta, Gamma, Kappa, Delta (India, lineage B.1.617.2, L452R, T478K), Lambda (Peru, lineage C37, L452Q, F490S) and Mu variants (Table S5). Note that CR3022 does not have contact with all R346, K417, L452, T478, E484, F490 and N501 residues of RBD, where the mutation is made for the aforementioned SARS-CoV-2 variants (Fig. S6). Therefore, we carried out SMD simulation only for H11-H4–RBD.

#### Beta, Gamma, Lambda and Mu variants reduce the binding affinity of H11-H4 to RBD

**Figure 5:**
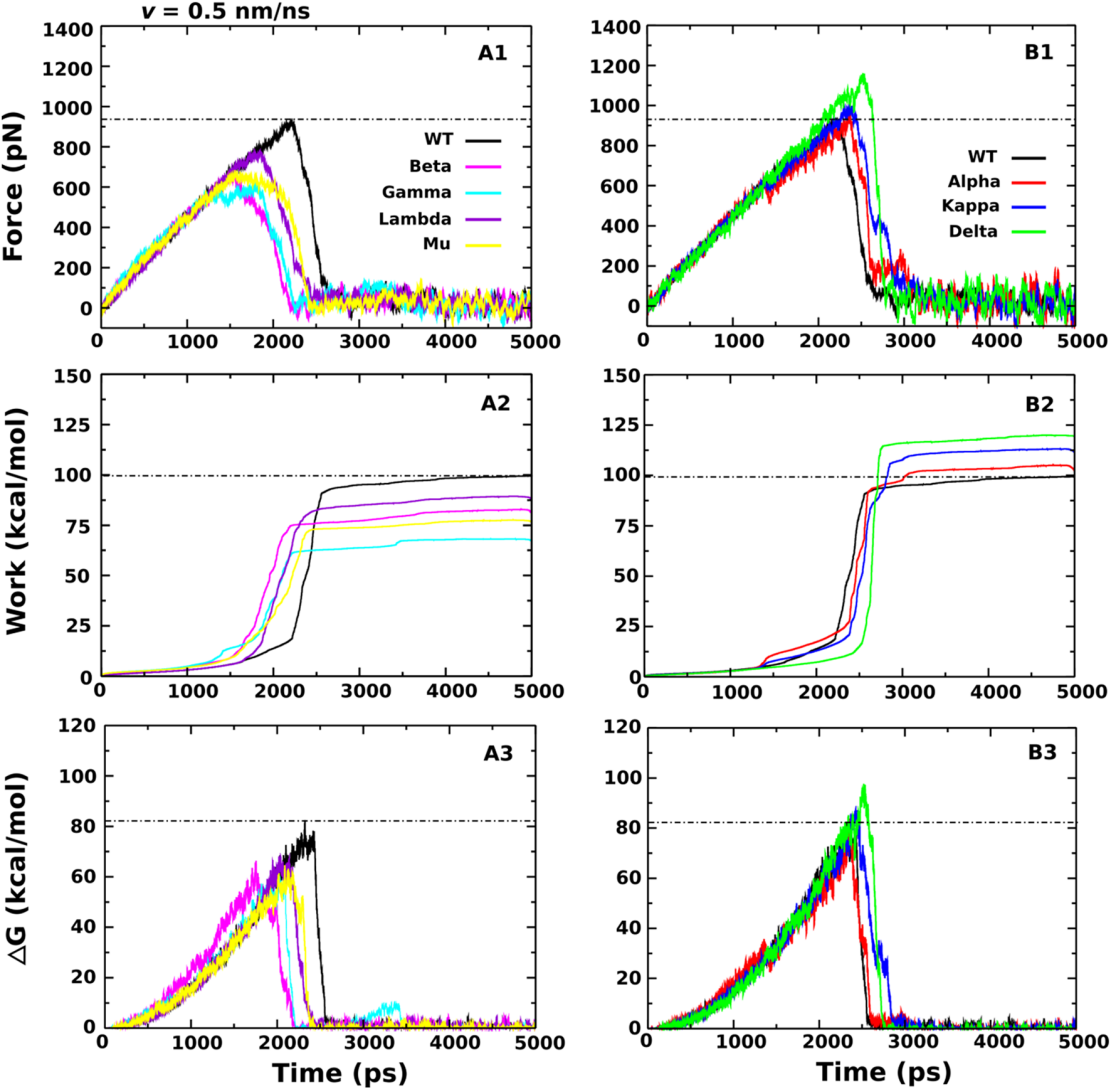
Time dependence of the force (**A1** and **B1**), work (**A2** and **B2**), and non-equilibrium free energy (**A3** and **B3**) of H11-H4-RBD. The results were obtained for WT and different variants at *v* = 0.5 nm/ns and averaged from five independent SMD runs.

As seen from Fig. 5A1–A3 and Table 4, *F*_max_, *W*, *∆∆G*_bind_ and *∆∆G*_unbind_ of Beta, Gamma, Lambda and Mu variants are lower than those of WT, suggesting that H11-H4 is less active against these variants. The decrease in the interaction between H11-H4 and RBD caused by the Beta, Gamma and Mu variants is mainly due to the E484K mutation, which increases the total non-bonded interaction energy at this position from − 156.3 kcal/mol (WT) to 62.9, 72.2 and 79.2 kcal/mol for Beta, Gamma and Mu variants, respectively (Table 5). The strong attractive interaction becomes even repulsive after E484K mutation.

**Table 4:** Rupture force (*F*_max_), pulling work (*W*), and non-equilibrium binding (*∆G*_bind_) and unbinding (*∆G*_unbind_) free energies obtained from five independent SMD trajectories with *v* = 0.5 nm/ns for H11-H4–RBD.

	WT	Beta (B.1.351)	Gamma (P.1)	Lambda (C37)	Mu (B.1.621)	Alpha (B.1.1.7)	Kappa (B.1.617.1)	Delta (B.1.617.2)
F_max_ (pN)	925.6 ± 42.2	714.5 ± 36.1	621.3 ± 38.7	776.2 ± 41.3	670.5 ± 33.1	982.6 ± 47.7	1018 ± 57.9	1162.1 ± 52.3
W (kcal/mol)	101.6 ± 3.3	80.8 ± 4.5	67.1 ± 4.9	87.4 ± 5.1	76.6 ± 6.0	105.8 ± 5.8	111.3 ± 9.3	119.5 ± 7.7
*∆*∆G_unbind_ (kcal/mol)	82.5 ± 2.1	66.4 ± 3.6	61.3 ± 4.1	68.1 ± 4.4	64.6 ± 3.3	83.8 ± 3.1	87.5 ± 7.0	97.6 ± 6.2
*∆*∆G_bind_ (kcal/mol)	82.1 ± 2.9	65.9 ± 4.1	58.7 ± 3.7	67.8 ± 4.8	64.2 ± 3.4	82.7 ± 3.6	85.8 ± 7.3	97.3 ± 5.4

Results are shown for WT and variants Alpha, Beta, Gamma, Kappa, Delta, Lambda and Mu. The errors represent standard deviations.

**Table 5:** The interaction energy (kcal/mol), which is the sum of the electrostatic and vdW interaction energy, between the important residues of RBD and H11-H4 in WT and different variants.

WT	Beta (B.1.351)	Gamma (P.1)	Lambda (C37)	Mu (B.1.621)	Alpha (B.1.1.7)	Kappa (B.1.617.1)	Delta (B.1.617.2)
R346: 71.5				K346: 62.8			
K417: 0	N417: 0	T417: 0					
L452: − 0.2			Q452: − 0.7			R452: 58.6	R452: 56.5
T478: 0							K478: 0
E484: − 156.3	K484: 62.9	K484: 72.2		K484: 79.2		Q484: − 20.1	
F490: − 8.4			S490: − 0.1				
N501: 0	Y501: 0	Y501: 0		Y501: 0	Y501: − 0.1		

The results were obtained in a [0, t_max_] time window and averaged from five SMD trajectories performed at a pulling speed of *v* = 0.5 nm/ns. Black and red refer to WT and mutations, respectively.

Meanwhile, the decrease in the interaction of H11-H4 with the Lambda variant occurs predominantly due to L452Q and F490S, which change the total non-bonded interaction energy from − 0.2 to − 8.4 kcal/mol (WT) to − 0.7 and − 0.1 kcal/mol (Lambda variant) (Table 5). The residues K417N-T and N501Y belong to the Beta, Gamma and Mu (only N501Y) variants, but do not interact with H11-H4. The R346K mutation reduced the total non-bonded interaction energy at this point from 71.5 (WT) to 62.8 kcal/mol (Mu variant) (Table 5), but this gain is not enough to compensate for the loss due to the E484K mutation for the Mu variant.

#### Alpha, Kappa and Delta variants increase the binding affinity of H11-H4 to RBD

In addition to the Beta, Gamma, Lambda and Mu variants, we also examined the binding affinity of H11-H4 to Alpha, Kappa and Delta variants. Unlike Beta, Gamma, Lambda and Mu variants, the *F*_max_, *W*, *∆∆G*_bind_ and *∆∆G*_unbind_ of Alpha, Kappa and Delta variants increase (Fig. 5B1–B3; Table 4), implying that H11-H4 can neutralize these variants better than WT.

For the Alpha variant, although the mutation point N501Y does not significantly contribute to the stability of H11-H4–RBD (Table 5), the binding affinity is insignificantly stronger than that of WT (Fig. 5B1–B3; Table 4). The total non-bonded interaction energy of N501Y slightly drops from 0 (WT) to − 0.1 kcal/mol. For the Kappa variant, the E484Q mutation destabilizes the H11-H4–RBD complex, as the corresponding total non-bonded interaction energy increases from − 156.3 (WT) to − 20.1 kcal/mol (Table 5). The L452R mutation also weakens the interaction with H11-H4 due to an increase in total non-bonded interaction energy from − 0.2 (WT) to 58.6 kcal/mol (Table 5). Based on the total non-bonded interaction energy obtained at mutation positions 484 and 452, we cannot explain why the Kappa variant enhances the stability of H11-H4–RBD complex. Same as the Kappa variant, the L452R mutation of the Delta variant has an increase in the total non-bonded interaction energy from − 0.2 (WT) to 56.5 kcal/mol (Table 5), but the binding affinity is still much higher than WT. So what is the reason for the increased binding affinity between H11-H4 and RBD in the Kappa and Delta variants?

**Figure 6:**
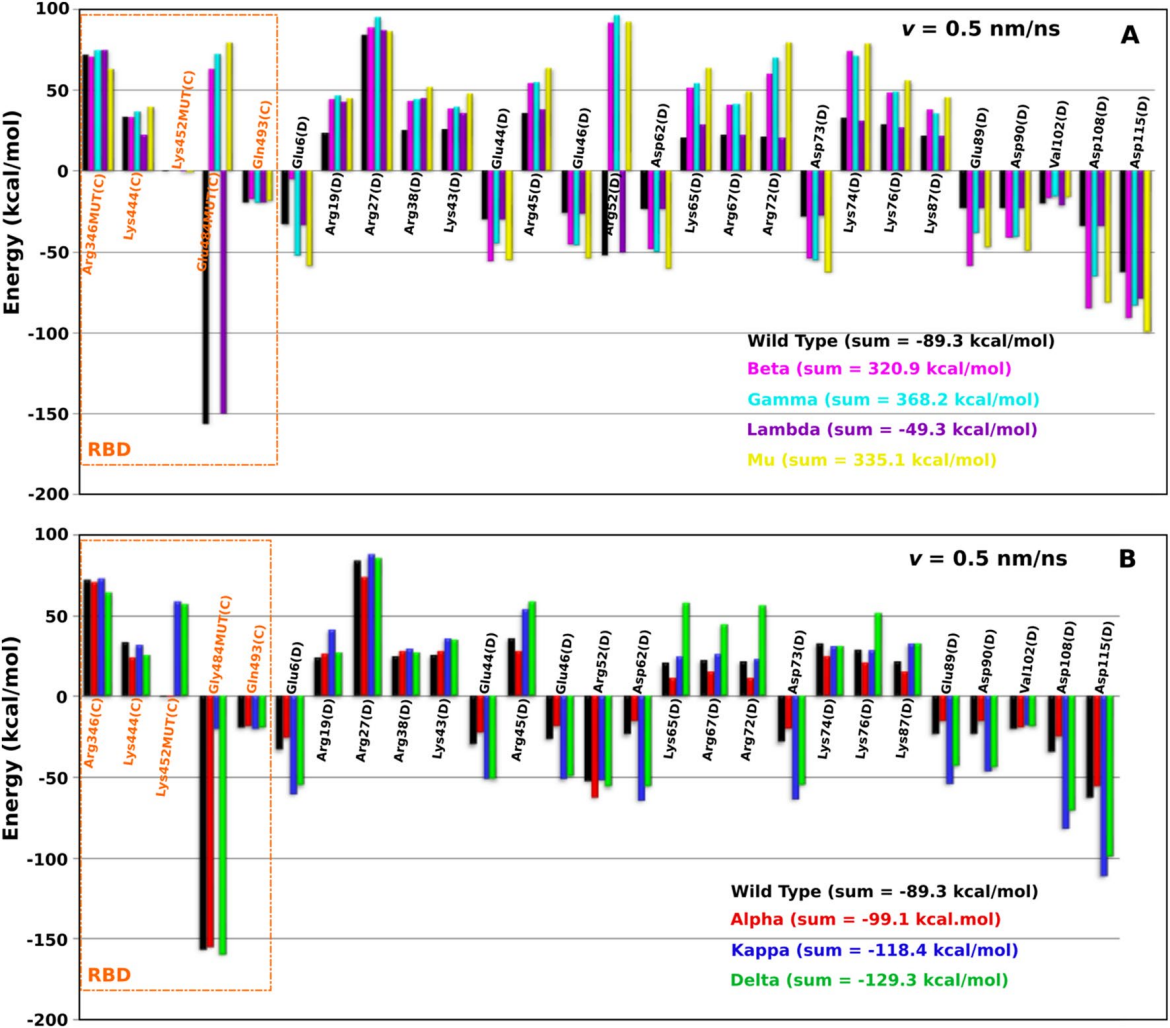
Total non-bonded interactions of the important residues of (**A**) H11-H4-RBD of WT and variants Beta, Gamma, Lambda and Mu, and (**B**) H11-H4-RBD of WT and variants Alpha, Kappa and Delta. The results were obtained in the time window [0, t_max_] and averaged from five independent SMD runs at a pulling speed of *v* = 0.5 nm/ns.

To solve these issues, we calculated the total interaction energy not only for the residues related to the mutation points, but also for all important residues (Fig. 6A,B). For WT, the total energy is − 89.3 kcal/mol, which is higher than Alpha (− 99.1 kcal/mol), Kappa (− 118.4 kcal/mol) and Delta (− 129.3 kcal/mol). For Gamma, Mu, Beta and Lambda we obtained 368.2, 335.1, 320.9 and − 49.3 kcal/mol, respectively, which is clearly higher than for WT. Therefore, the order of stability is as follows: Gamma < Mu < Beta < Lambda < WT < Alpha < Kappa < Delta. This finding is consistent with a report that nanobodies elicited from a llama could neutralize the Delta variant^41^. In addition, we also predict that H11-H4 maybe an excellent candidate to treat Alpha and Kappa variants.

### Binding free energy of H11-H4 and CR3022 to RBD: coarse-grained umbrella sampling results

The SMD, known as a method used to investigate the unbinding process of a small molecule to other molecules, is capable of predicting relative binding affinity but cannot be used to calculate the binding free energy. Overall, although the SMD method has provided a good correlation with experimental results^26,52,55,75^, their predictions are not always perfect. Therefore, we also used coarse-grained umbrella sampling to determine the binding free energies in an effort to elucidate the interactions of H11-H4–RBD, CR3022–RBD and H11-H4 + CR3022–RBD complexes.

**Figure 7:**
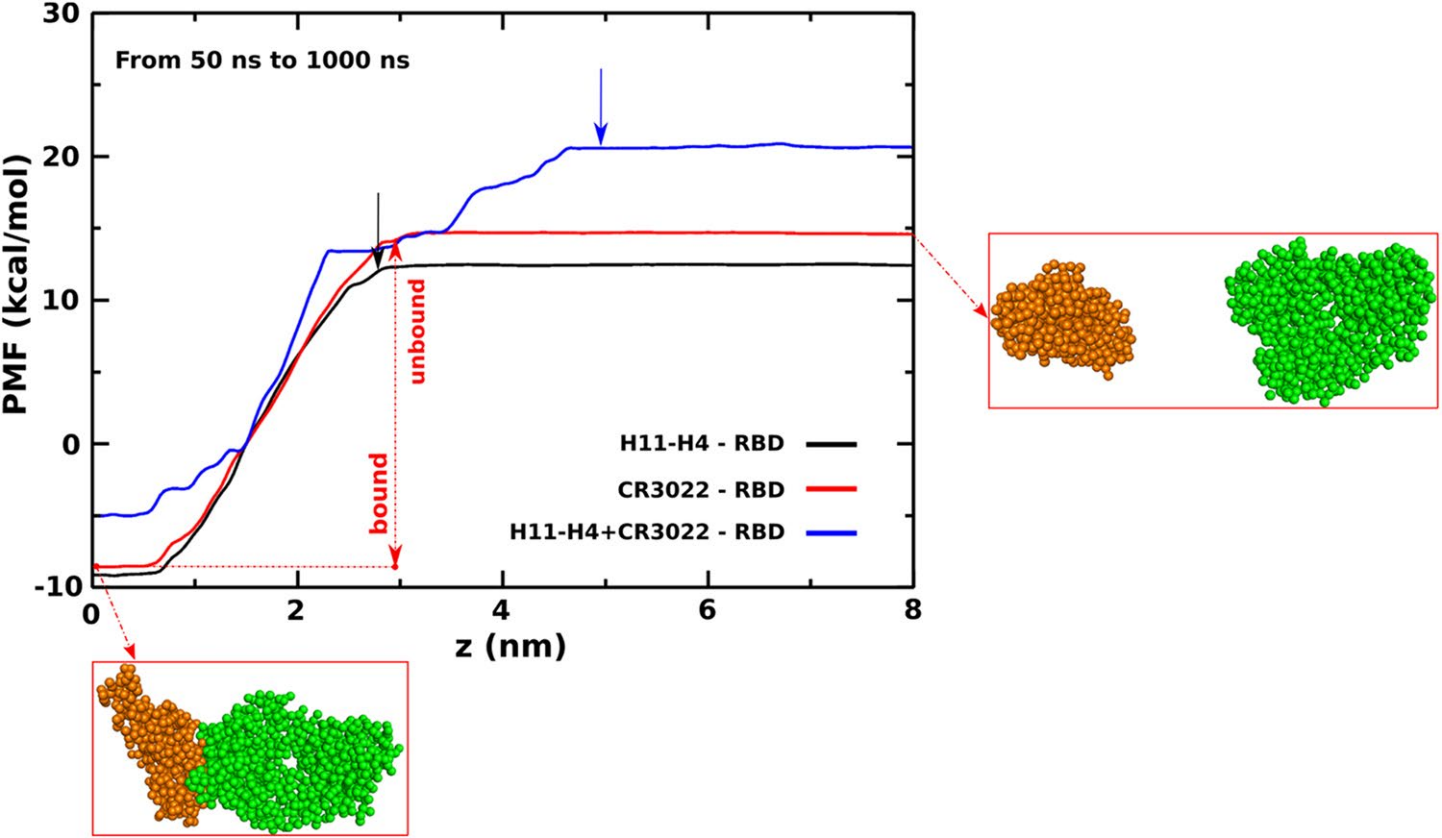
One-dimensional potential of mean force (1D PMF) of complexes H11-H4–RBD, CR3022–RBD and H11-H4 + CR3022-RBD as a function of the reaction coordinate. The result was obtained for a [50, 1000 ns] time window using CG-US simulations with the MARTINI force field. The left and right snapshots refer to the bound and unbound state of CR3022–RBD. The arrow indicates the position of the cutoff distance between bound and unbound states.

The MARTINI CG-US was used to estimate the binding free energy (*∆G*_bind_). To show that the equilibrium phase has been reached, we calculated the 1D PMF for three time intervals of 500, 800 and 1000 ns. Since the 1D PMF profiles for these windows are essentially the same (Fig. S7) our data was equilibrated. Therefore, the profile obtained from the largest window (Fig. 7) was used for estimating the binding free energy.

In order to use Eq. (4) to extract the binding free energy from the 1D PMF profiles we must estimate the cutoff distance between bound and unbound states, which was defined in “Material and methods”. From the distance dependence of the number of interchain contacts (Fig. S2) we obtained the cutoff distance of 4.9, 2.8 and 3.0 nm for H11-H4 + CR3022-RBD, H11-H4–RBD and CR3022–RBD, respectively. Using these cutoff distances, Eq. (4) and the 1D PMF profiles shown in Fig. 7, we obtained *∆G*_bind_ = − 19.8 kcal/mol for H11-H4–RBD and − 21.4 kcal/mol for CR3022-RBD (Table 1). The low value of *∆G*_bind_ of CR3022 indicates that this antibody tightly binds to the S protein which is consistent with previous computational studies^26^. Moreover, in agreement with the SMD results shown above and the in vitro results reported by Huo et al*.*^25^ (K_d_ = 11.8 nM, *∆G*_bind_ = − 10.9 kcal/mol for H11-H4) and Tian et al*.*^20^ (K_d_ = 6.3 nM, *∆G*_bind_ = − 11.3 kcal/mol for CR3022), H11-H4 binds to RBD weaker than CR3022. Obviously, the *∆G*_bind_ value obtained with the MARTINI CG-US is much lower than the experimental data, which may be related to the force field we used and the complexity of the studied systems.

According to our CG-US results, the relative binding affinity of CR3022 and H11-H4 is *∆G*_bind_(H11-H4)/*∆G*_bind_(CR3022) = − 19.8/− 21.4 = 0.93, which is not too far from experimental value *∆G*_exp_(H11-H4)/*∆G*_exp_(CR3022) = − 10.9/− 11.3 = 0.97. Thus, although the difference between simulation and experiment in absolute binding free energy is quite large, agreement on the relative binding affinity is satisfactory. In addition, bearing in mind that it is very challenging to obtain the absolute binding free energy even for small ligands using different MD based methods, our results are reasonable.

For H11-H4 + CR3022–RBD we obtained *∆G*_bind_ = − 23.9 kcal/mol, which shows that, consistent with the SMD results, the combination of antibody and nanobody enhances their binding affinity. Since *∆G*_bind_ (H11-H4 + CR3022)-*∆G*_bind_(CR3022) = − 23.9 − (− 21.4) = − 2.5 kcal/mol, we predict that in combination with H11-H4 the dissociation constant K_D_ of CR3022 is decreased by about 66 times (exp(2.5 kcal/mol/*RT*) ≈ 66). On the other hand, *∆G*_bind_ (H11-H4 + CR3022)-*∆G*_bind_(H11-H4) = − 23.9 − (− 19.8) = − 4.1 kcal/mol, which results in a decrease of K_D_ of H11-H4 by 960 times (exp(4.1 kcal/mol/*RT*) ≈ 960). Although these theoretical estimates are approximate, they show that the combination of antibody and nanobody significantly improves the neutralization of SARS-CoV-2 activity.

## Discussion and conclusion

Combining various computational methods, we studied the association of H11-H4, CR3022, and both H11-H4 and CR3022 with RBD. A number of interesting results have been obtained. SMD simulation showed that the H11-H4 nanobody binds to RBD weaker than CR3022, which is consistent with the binding free energy *∆G*_bind_ computed from the coarse-grained US. Our theoretical estimates of the binding affinity are in good agreement with the experimental results presented by Tian et al*.*^20^ and Huo et al*.*^25^ for H11-H4 and CR3022 interacting with SARS-CoV-2, but for the CR3022–RBD complex they contradict Yuan et al.^21^ (Table 1). To clarify the contradiction, recall that Tian et al*.*^20^ reported a *K*_d_ of 6.3 nM for CR3022–RBD, which is lower than Kd = 115 nM of Yuan et al*.*^21^ (Table 1). The difference in binding affinity may be due to differences in experimental conditions of these two groups, as discussed by Yuan et al*.*^21^. Namely, CR3022 was expressed as an Fab in Yuan et al*.*^21^, but single-chain fragment variable (scFv) in Tian et al*.*^20^; CR3022 was expressed in mammalian cells in Yuan et al*.*^21^, but in *E. coli* in Tian et al*.*^20^. SARS-CoV-2 RBD was expressed in insect 150 cells in Yuan et al*.*^21^, but in mammalian cells in Tian et al*.*^20^. Since the result of Yuan et al*.*^21^ does not agree with our simulations, we assume that their experimental conditions do not match our modeling. However, we cannot confirm this with molecular simulations because none of the existing models is able to capture differences in the behavior of proteins in different cells.

We predict that the concurrent binding of H11-H4 and CR3022 to RBD results in a higher binding affinity than when they are individually associated with RBD. Thus, the combination of H11-H4 and CR3022 enhances the neutralization of SARS-CoV-2, and this could open up a new treatment strategy for Covid-19. Whether this conclusion holds for the other antibody–nanobody pairs is a matter of further clarification.

Stability of the H11-H4–RBD complex is mainly contributed by the vdW interaction, while electrostatic interaction is more important for the CR3022–RBD and H11-H4 + CR3022–RBD complexes. In general, the role of vdW and electrostatic interaction in the binding of antibodies and nanobodies to SARS-CoV-2 depends on the specific case.

Our computational study found CR3022 to be a better candidate for treating Covid-19 than H11-H4, but only for WT. It is important to note that H11-H4 shows a high ability to neutralize the Alpha, Kappa and the highly dangerous Delta variants, and this fact is consistent with recent experiment.

In our work, the advantage of SMD is that it can provide all-atom description, but the disadvantage is that it does not allow the calculation of the free energy at equilibrium. In contrast, the equilibrium binding free energy can be obtained by coarse-grained umbrella sampling, but only at the coarse level. Nevertheless, these two approach complement each other, leading to a satisfactory description of the experiment.

For protein–ligand systems, SMD has been shown to be as efficient as the MM-PBSA method but computationally faster due to fast pull^76^. Although a similar analysis has not been carried out for protein–protein interactions, in conjunction with previous woks^26,55,75^, the present study shows that this method is useful for characterizing the relative binding affinity of protein–protein complexes. It would be useful to compare the SMD method with the MM-PBSA and other MD-based methods for estimating the absolute binding free energy of these complexes.

In the reaction coordinate space, PMF shown in Fig. 7 is one-dimensional (1D), and mapping the multidimensional free energy landscape to the 1D profile is an approximation. However, Z in Fig. 7 is the radial distance in real 3D space, which can reflect the 3D nature of the problem. This may be one of the reasons why umbrella sampling is one of the best methods for calculating free energy^77^. In other words, 1D PMF is adequate for our problem.

## Supplementary Information

Supplementary Information 1.

Supplementary Information 2.

## Data Availability

The data files are available in the “SciRep_data.zip” file, which includes: *SMD simulation data:* They are located in the “SMD_data” folder. The “WT” subfolder contains binding affinity and interaction energy data obtained from SMD simulation for the wild type. The “MUTATION” subfolder contains data on Covid-19 variants. *Coarse-grained simulation data*: The data obtained from umbrella sampling coarse-grained simulations are presented in the “US-CG” folder.

## References

[1] Cohen J (2020). Antibodies may curb pandemic before vaccines. Science.

[2] Cao X (2020). COVID-19: Immunopathology and its implications for therapy. Nat. Rev. Immunol..

[3] Chen L (2020). Convalescent plasma as a potential therapy for COVID-19. Lancet Infect. Dis..

[4] Shen C (2020). Treatment of 5 critically ill patients with COVID-19 with convalescent plasma. JAMA.

[5] Tortorici MA, Veesler D (2019). Structural insights into coronavirus entry. Adv. Virus Res..

[6] Simmons G (2013). Proteolytic activation of the SARS-coronavirus Spike protein: Cutting enzymes at the cutting edge of antiviral research. Antiviral Res..

[7] Gallagher TM, Buchmeier MJ (2001). Coronavirus Spike proteins in viral entry and pathogenesis. Virology.

[8] Belouzard S (2009). Activation of the SARS coronavirus Spike protein via sequential proteolytic cleavage at two distinct sites. Proc. Natl. Acad. Sci. USA.

[9] Wrapp D (2020). Cryo-EM structure of the 2019-nCoV Spike in the prefusion conformation. Science.

[10] Walls AC (2020). Structure, function, and antigenicity of the SARS-CoV-2 Spike glycoprotein. Cell.

[11] Krempl C (1997). Point mutations in the S protein connect the sialic acid binding activity with the enteropathogenicity of transmissible gastroenteritis coronavirus. J. Virol..

[12] Kunkel F, Herrler G (1996). Structural and functional analysis of the S proteins of two human coronavirus OC43 strains adapted to growth in different cells. Arch. Virol..

[13] Lu G (2015). Bat-to-human: Spike features determining ‘host jump’ of coronaviruses SARS-CoV, MERS-CoV, and beyond. Trends Microbiol..

[14] Zhou H (2019). Structural definition of a neutralization epitope on the N-terminal domain of MERS-CoV Spike glycoprotein. Nat. Commun..

[15] Tai W (2020). Characterization of the receptor-binding domain (RBD) of 2019 novel coronavirus: Implication for development of RBD protein as a viral attachment inhibitor and vaccine. Cell. Mol. Immunol..

[16] Lan J (2020). Structure of the SARS-CoV-2 Spike receptor-binding domain bound to the ACE2 receptor. Nature.

[17] Jiang S (2021). Therapeutic antibodies and fusion inhibitors targeting the spike protein of SARS-CoV-2. Expert Opin. Ther. Targets..

[18] Chen J (2021). Review of Covid 19 antibody therapies. Annu. Rev. Biophys..

[19] Lu M (2020). Real-time conformational dynamics of SARS-CoV-2 Spikes on virus particles. Cel Host Microbe.

[20] Tian X (2020). Potent binding of 2019 novel coronavirus Spike protein by a SARS coronavirus-specific human monoclonal antibody. Emerg. Microbes Infect..

[21] Yuan M (2020). A highly conserved cryptic epitope in the receptor binding domains of SARS-CoV-2 and SARS-CoV. Science.

[22] Jovčevska I, Muyldermans S (2020). The therapeutic potential of nanobodies. BioDrugs.

[23] Zhou P (2020). A pneumonia outbreak associated with a new coronavirus of probable bat origin. Nature.

[24] Wrapp D (2020). Structural basis for potent neutralization of Beta coronaviruses by single-domain camelid antibodies. Cell.

[25] Huo J (2020). Neutralizing nanobodies bind SARS-CoV-2 spike RBD and block interaction with ACE2. Nat. Struct. Mol. Biol..

[26] Nguyen H (2021). Electrostatic interactions explain the higher binding affinity of the CR3022 antibody for SARS-CoV-2 than the 4A8 antibody. J. Phys. Chem. B..

[27] Carolina CG (2020). On the interaction of the receptor-binding domain of SARS-CoV-1 and SARS-CoV-2 spike proteins with monoclonal antibodies and the receptor ACE2. Virus Res..

[28] Davies NG (2021). Estimated transmissibility and impact of SARS-CoV-2 lineage B.1.1.7 in England. Science.

[29] Karim SSA (2021). New SARS-CoV-2 variants—Clinical, public, health, and vaccine implications. N. Engl. J. Med..

[30] Faria NR (2021). Genomics and epidemiology of the P.1 SARS-CoV-2 lineage in Manaus, Brazil. Science.

[31] Singh J (2021). SARS-CoV-2 variants of concern are emerging in India. Nat. Med..

[32] Mlcochova P (2021). SARS-CoV-2 B.1.617.2 Delta variant replication and immune evasion. Nature.

[33] Kimura I (2022). SARS-CoV-2 Lambda variant exhibits higher infectivity and immune resistance. Cell Rep..

[34] Laiton-Donato K (2021). Characterization of the emerging B.1.621 variant of interest of SARS-CoV-2. Infect. Genet. Evol..

[35] Salvatore, M. *et al*. Resurgence of SARS-CoV-2 in India: Potential role of the B.1.617.2 (Delta) variant and delayed interventions. *medRxiv* (2021).

[36] Bai C (2021). Predicting mutational effects on receptor binding of the Spike protein of SARS-CoV-2 variants. J. Am. Chem. Soc..

[37] Jangra S (2021). SARS-CoV-2 skipe E484K mutation reduces antibody neutralisation. Lancet Microbe.

[38] Cheng, M. H. *et al*. Impact of South African 501.V2 variant on SARS-Cov-2 spike infectivity and neutralization: A structure-based computational assessment. *BioRxiv* (2021).

[39] Planas D (2021). Reduced sensitivity of SARS-CoV-2 variant Delta to antibody neutralization. Nature.

[40] Tada, T. *et al*. SARS-CoV-2 Lambda variant remains susceptible to neutralization by mRNA vaccine-elicited antibodies and convalescent serum. *BioRxiv* (2021).

[41] Xu J (2021). Nanobodies from camelid mice and llamas neutrlize SARS-CoV-2 variants. Nature.

[42] Webb B, Sali A (2016). Comparative protein structure modeling using MODELLER. Curr. Protoc. Bioinform..

[43] The PyMOL molecular graphics system, version 2.0 Schrödinger, LLC.

[44] Robustelli P (2018). Developing a molecular dynamics force field for both folded and disordered protein states. Proc. Natl. Acad. Sci. USA.

[45] Abraham MJ (2015). GROMACS: High performance molecular simulations through multi-level parallelism from laptops to supercomputers. SoftwareX.

[46] Bussi G (2007). Canonical sampling through velocity rescaling. J. Chem. Phys..

[47] Parrinello M (1981). Polymorphic transitions in single crystals: A new molecular dynamics method. J. Appl. Phys..

[48] Jorgensen WL, Jenson C (1998). Temperature dependence of TIP3P, SPC, and TIP4P water from NPT Monte Carlo simulations: Seeking temperatures of maximum density. J. Comput. Chem..

[49] Hess B (1997). LINCS: A linear constraint solver for molecular simulations. J. Comput. Chem..

[50] Darden T (1993). Particle mesh Ewald: An Nlog(N) method for Ewald sums in large systems. J. Chem. Phys..

[51] Hockney RW (1974). Quiet high-resolution computer models of a plasma. J. Comput. Phys..

[52] Nguyen H (2018). Steered molecular dynamics for investigating the interactions between Insulin Receptor Tyrosine Kinase (IRK) and variants pf Protein Tyrosine Phosphatase 1B (PTP1B). Appl. Biochem. Biotechnol..

[53] Nguyen H (2018). Binding affinity of the L-742,001 inhibitor to the endonuclease domain of A/H1N1/PA influenza virus variants: Molecular simulation approaches. Chem. Phys..

[54] Pham T (2020). Investigation of binding affinity between potential antiviral agents and PB2 protein of influenza A: Non-equilibrium molecular dynamics simulation approach. Int. J. Med. Sci..

[55] Nguyen HL (2020). Does SARS-CoV-2 bind to human ACE2 more strongly than does SARS-CoV?. J. Phys. Chem. B..

[56] Binnig G, Quate CF (1986). Atomic force microscope. Phys. Rev. Lett..

[57] Jarzynski C (1997). Nonequilibrium equality for free energy differences. Phys. Rev. Lett..

[58] Hummer G, Szabo A (2001). Free energy reconstruction from nonequilibrium single-molecule pulling experiments. Proc. Natl. Acad. Sci. USA.

[59] Park S (2003). Free energy calculation from steered molecular dynamics simulations using Jarzynski’s equality. J. Chem. Phys..

[60] Truong DT, Li MS (2018). Probing the binding affinity by Jarzynski’s nonequilibrium binding free energy and rupture time. J. Phys. Chem. B.

[61] Arnarez C (2015). Dry Martini, a coarse-grained force field for lipid membrane simulations with implicit solvent. J. Chem. Theory Comput..

[62] Monticelli L (2008). The MARTINI coarse-grained force field: Extension to proteins. J. Chem. Theory Comput..

[63] Uusitalo JJ (2017). Martini coarse-grained force field: Extension to RNA. Biophys. J..

[64] Yesylevskyy SO (2010). Polarizable water model for coarse-grained Martini force field. PLoS Comput. Biol..

[65] Berendsen HJC (1984). Molecular dynamics with coupling to an external bath. J. Chem. Phys..

[66] Thomas DP (2021). Automated coarse-grained mapping algorithm for the Martini force field and benchmarks for membrane-water partitioning. J. Chem. Theory Comput..

[67] Torrie GM, Valleau JP (1977). Nonphysical sampling distributions in Monte Carlo free-energy estimation: Umbrella sampling. J. Comput. Phys..

[68] Kumar S (1992). The weighted histogram analysis method for free-energy calculations on biomolecules. 1. The method. J. Comput. Chem..

[69] Patel JS, Ytreberg FM (2018). Fast calculation of protein-protein binding free energies using umbrella sampling with a coarse-grained model. J. Chem. Theory Comput..

[70] Wallace AC (1995). LIGPLOT: A program to generate schematic diagrams of protein-ligand interactions. Protein Eng..

[71] Evans E, Ritchie K (1997). Dynamic strength of molecular adhesion bonds. Biophys. J..

[72] Dudko OK (2006). Intrinsic rates and activation free energies from single-molecule pulling experiments. Phys. Rev. Lett..

[73] Vuong VQ (2015). A new method for navigating optimal direction for pulling ligand from binding pocket: Application to ranking binding affinity by steered molecular dynamics. J. Chem. Inf. Model..

[74] Pham HA (2021). Dependence of work on the pulling speed in mechaical ligand unbinding. J. Phys. Chem. B..

[75] Nguyen H (2022). Cocktail of REGN antibodies binds more strongly to SARS-CoV-2 than its components, but the Omicron variant reduces its neutralizing ability. J. Phys. Chem. B..

[76] Mai BK (2010). Top leads for swine influenza A/H1N1 virus revealed by steered molecular dynamics approach. J. Chem. Inf. Model..

[77] Kästner J (2011). Umbrella sampling. WIREs Comput. Mol. Sci..

