# Supplementary material for: Antibody–nanobody combination increases their neutralizing activity against SARS-CoV-2 and nanobody H11-H4 is effective against Alpha, Kappa and Delta variants
Source: Sci Rep. 2022 Jun 11;12:9701. doi: 10.1038/s41598-022-14263-1 (PMC9188278; doi:10.1038/s41598-022-14263-1)
Supplement: Supplementary file 1 — Supplementary Information 1. [file 41598_2022_14263_MOESM1_ESM.pdf]

Supporting Information

Antibody-nanobody combination increases their neutralizing activity against SARS-CoV-2 and nanobody H11-H4 is effective against Alpha, Kappa and Delta variants

Hung Nguyen[1] & **Mai Suan Li**[1,2,*]

[1]*Institute of Physics, Polish Academy of Sciences, al. Lotnikow 32/46, 02-668, Warsaw, Poland.*

[2]*Life Science Lab, Institute for Computational Science and Technology, Quang Trung Software City, Tan Chanh Hiep Ward, District 12, Ho Chi Minh City, Vietnam.*

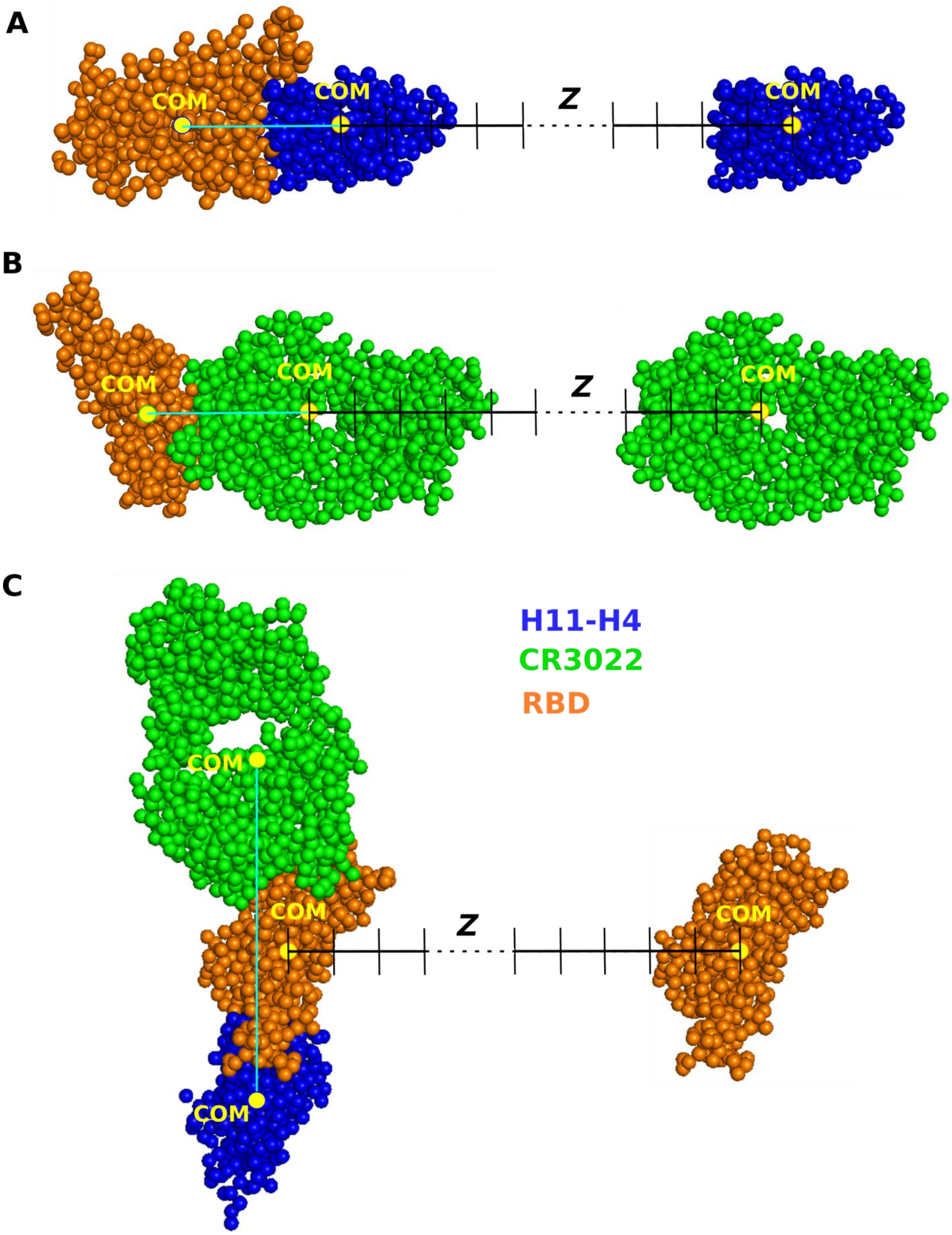

Figure S1. The reaction coordinate *z* used in the CG-US simulation. H11-H4 (blue), CR3022 (green) and RBD (orange) were pulled in this direction to generate a series of configurations separated by a distance of 0.1 nm. For H11-H4 – RBD (A) and CR3022 – RBD (B) *z* goes along the line connecting the two COMs. For H11-H4+CR3022 – RBD (C) z is perpendicular to the line connecting the COMs of antibody and nanobody.

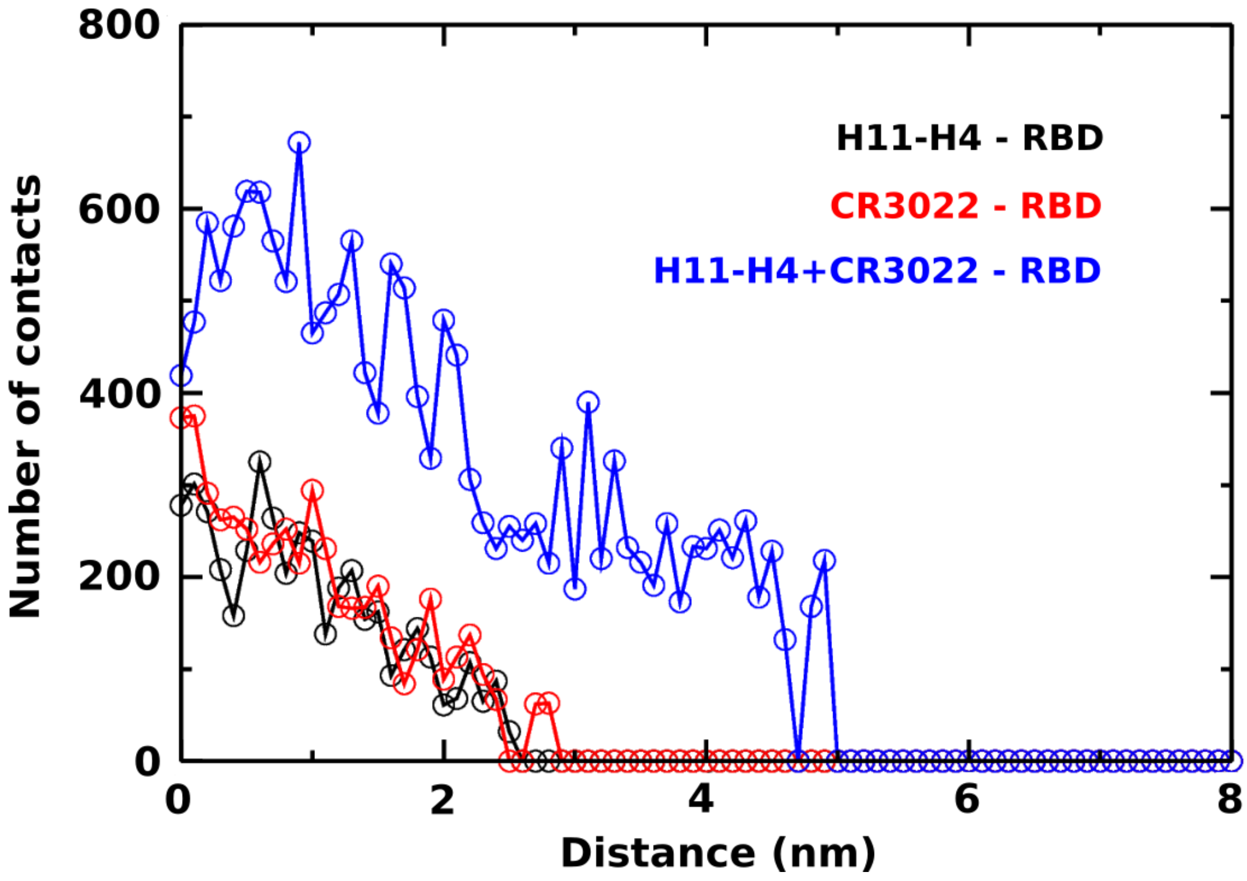

Figure S2. Determination of the cutoff distance separating bound and unbound states. Dependence of the number of interchain contacts on the distance *z* in the CG-US simulation. The number of contacts was calculated using the last snapshot of the 1000 ns trajectory for each CG-US window.

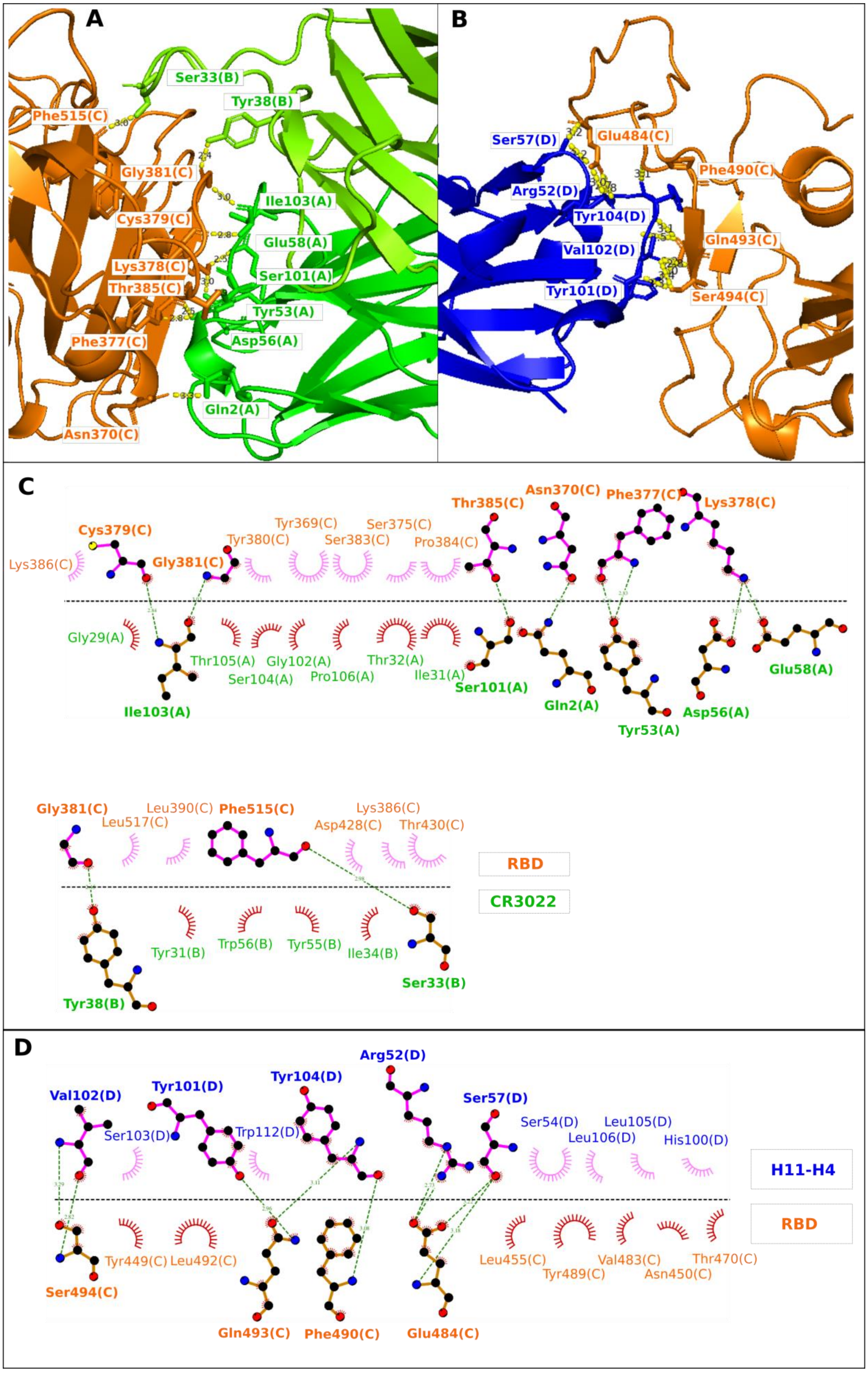
A
Ser33(B)
Tyr38(B)
Phe515(C)
Gly381(C)
Ile103(A)
Cys379(C)
Glu58(A)
Lys378(C)
Ser101(A)
Thr385(C)
Tyr53(A)
Asp56(A)
Phe377(C)
Gln2(A)
Asn370(C)
B
Ser57(D)
Glu484(C)
Arg52(D)
Phe490(C)
Tyr104(D)
Val102(D)
Gln493(C)
Tyr101(D)
Ser494(C)
C
Cys379(C)
Gly381(C)
Lys386(C)
Tyr380(C)
Tyr369(C)
Ser383(C)
Ser375(C)
Pro384(C)
Thr385(C)
Asn370(C)
Phe377(C)
Lys378(C)
Gly29(A)
Thr105(A)
Gly102(A)
Ser104(A)
Pro106(A)
Thr32(A)
Ile31(A)
Ser101(A)
Gln2(A)
Ile103(A)
Tyr53(A)
Asp56(A)
Glu58(A)
Gly381(C)
Leu517(C)
Leu390(C)
Phe515(C)
Asp428(C)
Lys386(C)
Thr430(C)
RBD
CR3022
Tyr31(B)
Trp56(B)
Tyr55(B)
Ile34(B)
Ser33(B)
Tyr38(B)
D
Val102(D)
Tyr101(D)
Tyr104(D)
Arg52(D)
Ser57(D)
Ser103(D)
Trp112(D)
Ser54(D)
Leu105(D)
Leu106(D)
His100(D)
H11-H4
RBD
Tyr449(C)
Leu492(C)
Leu455(C)
Tyr489(C)
Val483(C)
Asn450(C)
Thr470(C)
Ser494(C)
Gln493(C)
Phe490(C)
Glu484(C)

Figure S3. (A) Residues in the binding region of CR3022-RBD complex, the letter in parentheses refers to the chain name. (B) Same as in (A), but for the H11-H14 – RBD complex. (C) Networks of hydrogen bond and non-bonded contacts of CR3022 - RBD. (D) Same as in (C) but for H11-H4 - RBD. They were obtained by using the PDB structure with ID 6ZH9. Dotted line refers to HB, while the “eye” refers to NBC.

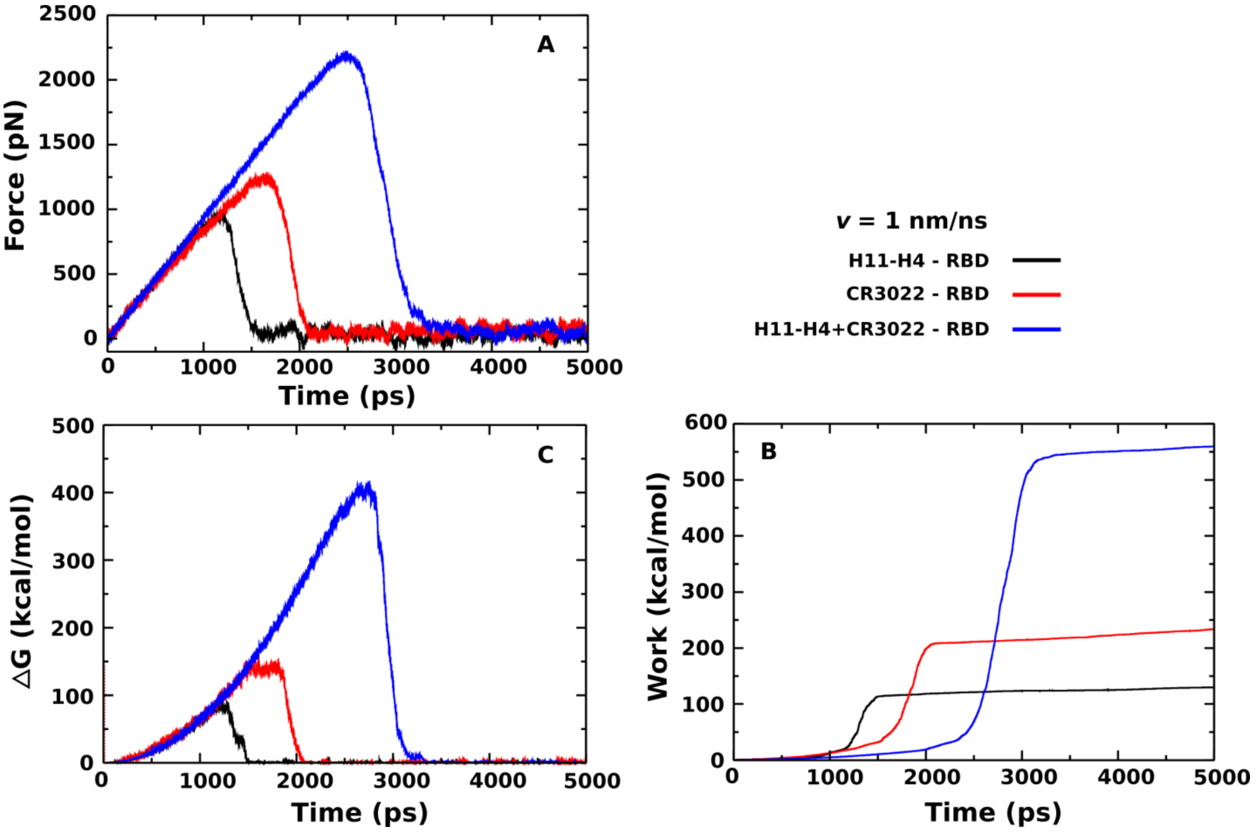

Figure S4: Time dependence of the pulling force (A), pulling work (B), and non-equilibrium free energy (C) of the H11-H4 - RBD, CR3022 - RBD, and H11-H4+CR3022 - RBD complexes. The results were obtained from five independent SMD runs at v = 1 nm/ns.

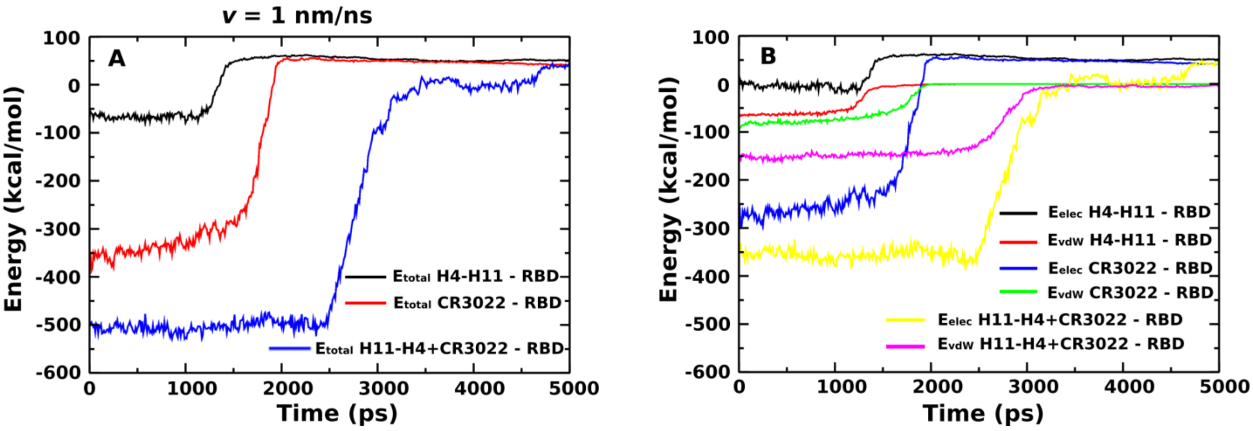

Figure S5. (A) The total non-bonded interaction (sum of electrostatic and vdW) and (B) electrostatic and vdW interactions of the H11-H4 - RBD, CR3022 - RBD, and H11-H4+CR3022 - RBD complexes as a function of SMD simulation time. The results were obtained from five independent SMD runs at pulling speed *v* = 1 nm/ns.

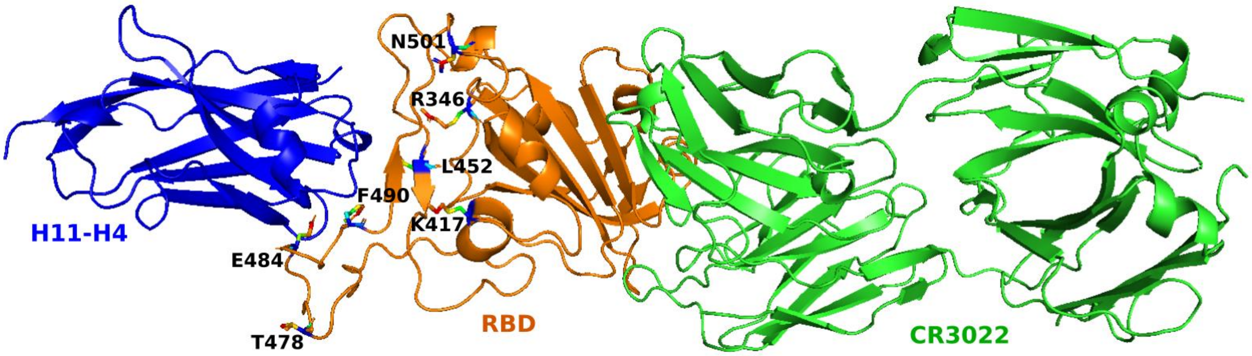

Figure S6. Mutations in RBD of the variants Alpha, Beta, Gamma, Kappa, Delta, Lambda and Mu. They have contact with H11-H4, but not with CR3022.

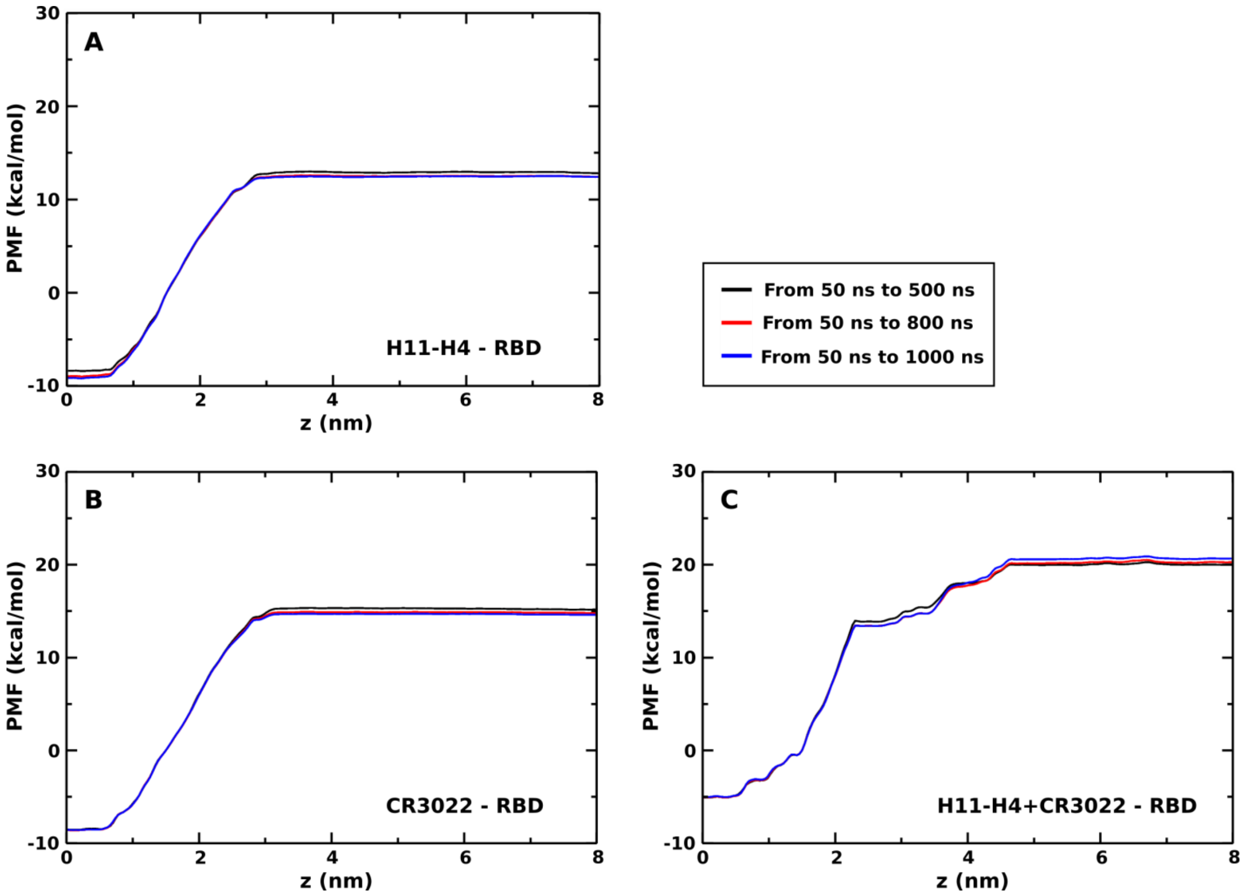

Figure S7. Illustration shows the potential of mean force (PMF) as a function of the reaction coordinate for (A) H11-H4 - RBD, (B) CR3022 - RBD, and (C) H11-H4+CR3022 - RBD complexes obtained for time windows [50, 500], [50, 800], and [50, 1000] ns. These results were obtained from CG-US simulations using the MARTINI force field.

Table S1. The rupture force (F_{max}), rupture time (t_{max}), work of external force (W), non-equilibrium binding (ΔG_{bind}) and unbinding (ΔG_{unbind}) free energies obtained from five independent SMD trajectories at pulling speed v = 1 nm/ns for H11-H4 - RBD, CR3022 - RBD, and H11-H4+CR3022 - RBD complexes.

	**v = 1 nm/ns**		
	H11-H4	CR3022	H11-H4+CR3022
**F_{max}**(pN)	970.7±14.0	1260.6±13.1	2197.9±17.4
**t_{max}**(ps)	1155.2±21.7	1608.8±25.5	2456.5±32.2
**W**(kcal/mol)	129.4±5.9	233.5±5.4	559.2±9.7
**$\Delta\Delta G_{unbind}$**(kcal/mol)	88.3±3.1	155.3±4.2	412.8±3.3
**$\Delta\Delta G_{bind}$**(kcal/mol)	87.9±4.3	153.5±5.1	412.6±4.1

Table S2. Non-bonded interaction energy (kcal/mol) of H11-H4 - RBD, CR3022 - RBD, and H11-H4+CR3022 - RBD complexes. The results were obtained for a [0, t_{max}] time window and averaged from five SMD trajectories performed at pulling speed v = 1 nm/ns.

**Energy**	**v = 1 nm/ns**		
	H11-H4	CR3022	H11-H4+CR3022
**E_{vdW}**	-64.7±1.6	-75.5±0.6	-147.5±0.4
**E_{elec}**	-1.7±0.3	-253.7±1.6	-355.6±0.7
**E_{total}**	-66.4±1.9	-329.2±2.2	-503.1±1.1

Table S3. Energy of electrostatic and vdW interactions between antibodies and RBD (in kcal/mol). Results were obtained using the PDB structure and CHARMM36M force field with the TIP3P water model.

**PDB ID**	E_{vdW}	E_{elec}	E_{total}
6ZH9 (CR3022-RBD)	-75.5	-253.7	-329.2
7M3I (CV2-75-RBD)	-81.6	-349.5	-431.1
7CH5 (BD-629-RBD)	-82.2	-231.3	-313.5
6ZER (EY6A-RBD)	-80.5	-183.7	-264.2
7BZ5 (B38-RBD)	-109.3	-116.7	-226.0
6XC4 (CC12.3-RBD)	-81.4	-35.8	-117.2
6XKP (CV07-270-RBD)	-82.8	-21.8	-104.6
6XC2 (CC12.1-RBD)	-27.0	-0.9	-27.9
7CM4 (CT-P59-RBD)	-26.0	-0.1	-26.1
7CH4 (BD-604-RBD)	-21.0	-0.1	-21.1

Table S4. Energy of electrostatic and vdW interactions between nanobodies and RBD (in kcal/mol). Results were obtained using the PDB structure and CHARM36M force field with the TIP3P water model.

**PDB ID**	E_{vdW}	E_{elec}	E_{total}
7KN5 (VHH U-RBD)	-60.0	-387.6	-447.6
7NKT (NM1226-RBD)	-71.7	-276.9	-348.6
7JVB (Nb20-RBD)	-76.9	-164.8	-241.7
7KN5 (VHH E-RBD)	-61.1	-95.1	-156.2
7LX5 (WNb 10-RBD)	-84.6	-85.5	-169.1
6YZ5 (H11-D4-RBD)	-59.5	-25.8	-85.3
7ME7 (Nb17-RBD)	-68.7	-15.0	-83.7
7MEJ (Nb21-RBD)	-50.6	-10.1	-60.7
6ZH9 (H11-H4-RBD)	-64.7	-1.7	-66.4
7LX5 (WNb 2-RBD)	-12.0	-0.2	-12.2

Table S5. Mutations in RBD and NTD of Alpha (United Kingdom), Beta (South Africa), Gamma (Brazil), Kappa (India), Delta (India), Lambda (Peru) and Mu (Colombia) variants. The name of the lineage is also displayed.

United Kingdom, **Alpha** (B.1.1.7)		Δ69-70, Δ144	N501Y
South Africa, **Beta** (B.1.351)		D80A, D215G, Δ241-243	K417N, E484K, N501Y
Brazil, **Gamma** (P.1)		L18F, T20N, P26S, D138Y, R190S	K417T, E484K, N501Y
India	(B.1.617)		L452R, E484Q
	**Kappa** (B.1.617.1)	E154K	L452R, E484Q
	**Delta** (B.1.617.2)	T19R, G142GD, Δ156-157, R158G	L452R, T478K
	(B.1.617.3)	T19R, Δ156-157, R158G	L452R, E484Q
Peru, **Lambda** (C37)		RSYLTPGD246-253N, GT75-76VI	L452Q, F490S
Colombia, **Mu** (B.1.621)		T95I, Y144T, Y145S, ins146N	R346K, E484K, N501Y
